# Primary Tumor Sidedness Associated with Clinical Characteristics and Postoperative Outcomes in Colon Cancer Patients: A Propensity Score Matching Analysis

**DOI:** 10.3390/jcm13133654

**Published:** 2024-06-22

**Authors:** Wan-Hsiang Hu, Samuel Eisenstein, Lisa Parry, Sonia Ramamoorthy

**Affiliations:** 1 Department of Colorectal Surgery, Kaohsiung Chang Gung Memorial Hospital and Chang Gung University College of Medicine, Kaohsiung 833, Taiwan; gary.hu0805@gmail.com; 2 Graduate Institute of Clinical Medical Science, College of Medicine, Chang Gung University, Kaohsiung 333, Taiwan; 3 Department of Surgery, University of California, San Diego Health System, La Jolla, CA 92103, USA; seisenstein@mail.ucsd.edu (S.E.); lparry@mail.ucsd.edu (L.P.); 4 Rebecca and John Moores Cancer Center, University of California, San Diego Health System, La Jolla, CA 92103, USA

**Keywords:** sidedness, postoperative complication, colon cancer, propensity score matching

## Abstract

**Background:** Recent investigations have suggested that-sidedness is associated with the prognosis of colon cancer patients. However, the role of sidedness in surgical outcome is unclear. In this study, we tried to demonstrate the real role of sidedness in postoperative results for colon cancer patients receiving surgical intervention. **Methods:** This is a propensity score matching study using the database of the American College of Surgeons-National Surgical Quality Improvement Program (ACS-NSQIP) from 2009 to 2013. Sidedness groups including right-sided and left-sided colon cancer were created according to the associated diagnosis and procedure codes. Postoperative 30-day mortality, morbidity, overall complications, and total length of hospital stay were analyzed after performing propensity score matching. **Results:** Out of a total of 24,436 colon cancer patients who received associated operations, 15,945 patients had right-sided cancer and 8941 patients had left-sided cancer. Right-sided colon cancer patients were accompanied by more preoperative comorbidities including old age, female sex, hypertension, dyspnea, anemia, hypoalbuminemia, and a high American Society of Anesthesiologists grade (SMD > 0.1). Postoperative mortality, morbidities including re-intubation, bleeding, urinary tract infection and deep vein thrombosis, postoperative overall complications, and total length of hospital stay were significantly associated with right-sided cancer (*p* < 0.05). After 1:1 propensity score matching, postoperative mortality was not significantly different between right-sided cancer (2.3%) and left-sided cancer (2.4%) patients. The patients with left-sided colon cancer had significantly more postoperative morbidities, more overall complications, and longer total length of hospital stay. **Conclusions:** Poor clinical characteristics and postoperative outcomes were noted in right-sided cancer patients. After propensity score matching, left-sided cancer patients had worse postoperative outcomes than those with right-sided cancer.

## 1. Introduction

In the United States and a global cancer survey, the incidence of colorectal cancer ranks third among all cancers [1,2]. However, it is still the most popular cancer in Taiwan, although fecal occult blood tests have been promoted for many years [3]. It is necessary to demonstrate the predictive and prognostic markers for further precision treatment.

In the past several years, primary tumor sidedness has been recognized as the predictive value of prognosis of colon cancer patients. Right-sidedness was demonstrated to be associated with poor survival in the colon cancer patients with metastasis [4,5,6,7,8], stage III [9], stage II–III microsatellite stable [10], unresectable recurrence [11]. However, the difference between right-sided and left-sided colon cancer in surgical outcome has seldom been reported on and continues to be controversial [12,13,14].

The American College of Surgeons began to set up a large standards-based database, the National Surgical Quality Improvement Program (ACS-NSQIP), in 1994. The data in this database include multiple clinical characteristics, blood examinations, and postoperative complications of major operations and were recorded by more than five hundred medical institutes of the United States and Canada [15]. The value of these data has been nationally and multi-institutionally validated. The purpose of the ACS-NSQIP is to improve surgical quality and outcomes after evaluating and analyzing the abundant clinical information. The propensity score matching method is a kind of statistical tool used to decrease differences in clinical comorbidities between target group and control cohorts in a nonrandomized database [16]. After matching, the qualities as a clinical randomized trial is set up to avoid confounding factors. The results of propensity score matching help us to identify the real role of clinical risk factors affected by the other associated risk factors.

The previous studies focusing on the relationship between sidedness and postoperative outcomes were based on non-randomized cohorts. The results of postoperative outcomes can be affected by many different risk factors and thus are controversial. The object of this study was to demonstrate the real effect of sidedness on the clinical characteristics and postoperative outcomes of colon cancers patients based on a robust database using propensity score matching.

## 2. Materials and Methods

### 2.1. Study Design and Population

The database of the American College of Surgeons-National Surgical Quality Improvement Program (ACS-NSQIP) covering the years 2009 to 2013 was used to provide data on colon cancer patients undergoing associated operations based on ICD-9 (International Classification of Disease, Ninth Revision) diagnosis codes and Current Procedural Terminology (CPT) codes (Supplement S1). The patients with malignant neoplasms of the appendix, cecum, ascending colon, and transverse colon were grouped into the right-sided and left-sided groups, included splenic flexure, descending colon, and sigmoid colon cancer. The ICD-9 diagnosis codes were 153.0, 153.1, 153.4, and 153.6 for right-sided colon cancer and 153.2, 153.3, and 153.7 for left-sided colon cancer. The Current Procedural Terminology (CPT) codes of associated operations included partial colectomy or total colectomy with laparotomy or laparoscopic methods. The CPT codes were most commonly identified in the variable Principle Operative Procedure. The patients with the CPT codes recognized in the variables Other Procedure and Concurrent Procedure Record were also enrolled in our study. The criteria and definition of the data content were based on a user guide for the 2013 ACS-NSQIP participant use data file (Supplement S2). The database was deidentified and recorded no patient’s personal information. The database is considered exempt from human subject research, and thus did not need to be approved by the Institutional Review Board.

### 2.2. Postoperative Outcomes

We defined the first postoperative outcomes as 30-day postoperative mortality and sixteen morbidities after the operation. The second postoperative outcomes included overall complications consisting of 30-day postoperative mortality and the morbidities, as well as the total length of hospital stay. The sixteen morbidities after the operation were recorded as surgical site infection (superficial, deep, and organic), sepsis, septic shock, urinary tract infection (UTI), pneumonia, wound disruption, reintubation, ventilator >48 h, pulmonary embolism (PE), deep vein thrombosis (DVT), stroke, myocardial infarction, blood transfusion, and return to the operating room (OR). According to the Accordion Severity Grading System, mortality and all morbidities had individually weighted scores using individual grades [17,18]. The postoperative overall complication scores of each patient were computed as the sum of the total weighted scores of the morbidities. The total length of hospital stay was defined as the number of calendar days from hospital admission to discharge.

### 2.3. Statistical Analysis

**Figure 1:**
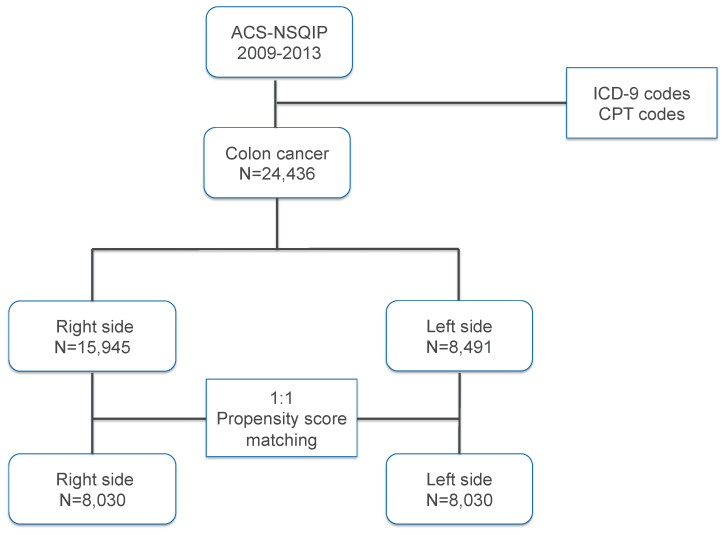
A flow chart of how to select and match the sided colon cancer patients.

In our study, left-sided colon cancer patients were defined as “control”, and the “case” cohort was defined as right-sided colon cancer patients. The propensity scores of the colon cancer patients were computed using a logistic regression tool according to the comorbidities, including twenty-three clinical characteristics and blood tests. These were age, gender, hypertension, dyspnea, American Society of Anesthesiologists (ASA) grade I–V, wound classifications II–IV, functional status (independent, partially dependent and totally dependent), diabetes mellitus (DM) (no, oral drug and insulin), chronic obstructive pulmonary disease (COPD), sepsis, smoking, disseminated cancer (DC), congestive heart disease (CHF), emergent operation, steroid treatment, body mass index (BMI) < 18.5, dialysis, ventilator, body weight loss (BWL), ascites, renal failure, anemia, and hypoalbuminemia. Wound classifications included clean/contaminated, contaminated and dirty/infected wounds. BMI was calculated as the body weight divided by the square of the height. BMI < 18.5 was defined as underweight according to World Health Organization criteria [19]. Anemia was defined as serum hematocrit < 38% [13]. The patients with hypoalbuminemia were identified as serum albumin levels < 3.5 g/dL. A 1:1 ratio propensity score matching for study groups was set up using the greedy method with a 0.2 caliper width using NCSS 10 software (NCSS Statistical Software, Kaysville, UT, USA) [20]. Standardized mean difference (SMD) was adopted to examine the balance of covariate distribution between the two cohorts [21]. The difference was significant when SMD was larger than 0.1. Figure 1 shows the flow chart of how we selected and matched the sided colon cancer patients. Univariate analysis was performed using the chi-square test method. We used the binary logistic regression method to demonstrate the significance of sidedness in 30-day mortality and morbidities after the operation. Regression analysis was performed to analyze the association among overall complication, days of total hospital stay, and sidedness. Tests were 2-tailed and statistical significance was demonstrated when the *p* valve was less 0.05. All of the statistical analyses were computed with SPSS for Windows version 22.

## 3. Results

Based on the data in the ACS-NSQIP, a total of 24,436 colon cancer patients who had received definite colectomy were studied according to ICD-9 diagnosis codes and CPT codes. They were divided into the right-sided colon cancer group (n = 15,945) and the left-sided colon cancer group (n = 8491). The differences in clinical characteristics between right-sided and left-sided colon cancer patients are shown in Table 1. Patients with right-sided colon cancer were more likely to be older, female, and have more comorbidities including hypertension, dyspnea, anemia, hypoalbuminemia, and high grade of American Society of Anesthesiologists (SMD > 0.1). More contamination and dirty incision wounds were classified in the patients receiving left colon cancer operations (SMD = 0.132). After 1:1 ratio propensity score matching, 8030 patients each with right-sided colon cancer or left-sided colon cancer were retained for comparison. No significant differences in the twenty-three clinical characteristics and blood tests were noted between the two groups after matching (Table 1).

Table 2 shows the difference in the incidence of morbidities after the operation between right-sided colon cancer and left-sided colon cancer patients before matching. Mortality and all significantly associated postoperative morbidities including reintubation, transfusion, urinary tract infection, and deep vein thrombosis were likely to occur in the patients with right-sided colon cancer (*p* > 0.05). After matching, postoperative mortality was not significantly different between right-sided cancer (2.3%) and left-sided cancer (2.4%). The only significantly different postoperative morbidities were myocardial infarction and stroke, and both were associated with the patients receiving left colon cancer operations (Table 3).

In the full cohort study, right-sided colon cancer patients were associated with postoperative overall complications and the total length of hospital stay using regression analyses. After propensity score matching, more overall complications and a significantly longer total length of hospital stay after the operation were noted in the patients with left-sided colon cancer than in those with right-sided colon cancer (Table 4).

## 4. Discussion

Using the multi-institutional and robust ACS-NSQIP database, poor postoperative outcomes were identified and associated with right-sided colon cancer patients, displaying more preoperative comorbidities than those with left-sided colon cancer. We used a propensity score matching tool to minimize the possible biases of clinical characteristics which were associated significantly with sidedness. After matching, postoperative outcomes including myocardial infarction, stroke, length of hospital stay, and overall complications were predominant in the patients with left-sided colon cancer. This is the first study to use propensity score matching to demonstrate the real differences in postoperative outcomes associated with sidedness.

According to our study, the average age of the patients diagnosed as having right-sided colon cancer was 70.8, which was significantly older than left-sided colon cancer patients (65). Women were predominant among the right-sided colon cancer patients. This relationship between sidedness, age, and gender was similar to previous reports [6,12,22,23]. The divergence of epidemiology caused the right-sided colon cancer patients to have more comorbidities, especially anemia and hypoalbuminemia, which was consistent with Kwaan’s finding [13].

In the published literature, right-sided colon cancer patients were associated with poor postoperative complications [12], a longer length of hospital stay [13], and mortality [22], which were also identified in our analysis before propensity score matching. Propensity score matching is a method widely used to create a balanced covariate distribution between the compared groups [21]. After matching, the patients with left-sided colon cancer were found to actually be associated with poor postoperative outcomes.

Comparisons between the outcomes of cetuximab and bevacizumab when added to first-line chemotherapy regimens for metastatic colorectal cancer patients with KRAS wild type were performed in the FIRE-3 [24] and CALGB 80405 [25] trials. After combining the impact of primary tumor location, patients with left-sided KRAS wild type (WT) colon cancer receiving cetuximab plus chemotherapy had significantly better survival and response rates than those receiving bevacizumab plus chemotherapy [26]. However, in Yin’s propensity score matching study, they demonstrated that no statistically significant treatment differences were present in patients with KRA WT left-sided colon cancer receiving the two kinds of combination regimens [8]. In Huang’s study, left-sided colon cancer patients had superior survival before matching, but no difference in survival was noted between left- and right-sided colon cancer patient in a matched cohort [27].

Recently, the molecular biomarkers including mutation of RAS, BRAF, and high microsatellite instability associated with colon carcinogenesis and poor prognosis were found to be more frequent in right-sided colon cancer patients [28,29]. A previous meta-analysis study demonstrated that a right-sided primary colon cancer location was associated with a significantly increased risk of death, and this was independent of some clinical characteristics, but not the covariates including comorbidities and molecular biomarkers [30]. The conclusion may be different if using propensity score matching to balance the distribution of covariates in the two-sided groups.

There are some limitations in our study. First, some of the specific postoperative complications after colectomy, for example, postoperative ileus and anastomotic leakage, were not recorded in the database. Second, the postoperative complications were only recorded during the 30-day postoperative period. Some of the postoperative complications that occurred after 30 days could not be estimated. Finally, the database did not include cancer-specific parameters, such as stage and tumor size, which may impact the postoperative outcomes. The poor effect of an advanced stage on postoperative outcomes would be minimized after excluding the patients who only received palliative operations.

## 5. Conclusions

Right-sided colon cancer patients are older, more commonly female, and had more comorbidities. After propensity score matching, right-sided colectomy was found to be associated with fewer postoperative complications and a shorter total length of hospital stay than left-sided colectomy. Further studies should include more risk factors and biomarkers to demonstrate the real role of sidedness in colon cancer patients’ prognosis.

## Figures and Tables

**Table 1:** Selected cohort comorbidities before and after propensity score matching.

Variables	Full Cohort			Matched Cohort (1:1)		
	Right Side (n = 15,945)	Left Side (n = 8491)	SMD	Right Side (n = 8030)	Left Side (n = 8030)	SMD
Age, mean (SD), y	70.8 (13)	65 (13.6)	0.433	65.9 (13.5)	66 (13.2)	0.001
Gender, female, n (%)	8646 (54.2)	4000 (47.1)	0.143	3811 (47.5)	3845 (47.9)	0.008
Hypertension, n (%)	9780 (61.3)	4633 (54.6)	0.138	4516 (56.2)	4483 (55.8)	0.008
Dyspnea			0.145			0.020
No	13627 (85.5)	7655 (90.2)		7249 (90.3)	7201 (89.7)	
Exertion	2109 (13.2)	749 (8.8)		702 (8.7)	747 (9.3)	
Rest	209 (1.3)	87 (1)		79 (1)	82 (1)	
Anemia	11336 (71.1)	4475 (52.7)	0.386	4443 (55.3)	4429 (55.2)	0.004
Hypoalbuminemia	5348 (33.5)	2368 (27.9)	0.123	2237 (27.9)	2289 (28.5)	0.014
ASA			0.197			0.014
I	221 (1.4)	224 (2.6)		177 (2.2)	189 (2.4)	
II	4980 (31.2)	3313 (39)		3074 (38.3)	3059 (38.1)	
III	9318 (58.4)	4329 (51)		4195 (52.2)	4178 (52)	
IV	1415 (8.9)	617 (7.3)		577 (7.2)	596 (7.4)	
V	11 (0.1)	8 (0.1)		7 (0.1)	8 (0.1)	
Wound			0.132			0.015
II	14,606 (91.6)	7439 (87.6)		7135 (88.9)	7098 (88.4)	
III	859 (5.4)	649 (7.6)		553 (6.9)	581 (7.2)	
IV	480 (3)	403 (4.7)		342 (4.3)	351 (4.4)	
Functional status, n (%)			0.075			0.017
Independent	14,907 (93.5)	8079 (95.1)		7654 (95.3)	7628 (95)	
Partially Dependent	893 (5.6)	341 (4)		317 (3.9)	333 (4.1)	
Totally Dependent	145 (0.9)	71 (0.8)		59 (0.7)	69 (0.9)	
Diabetes mellitus, n (%)			0.064			0.005
No	12,600 (79)	6918 (81.5)		6492 (80.8)	6495 (80.9)	
Oral	2274 (14.3)	1097 (12.9)		1064 (13.3)	1070 (13.3)	
Insulin	1071 (6.7)	476 (5.6)		474 (5.9)	465 (5.8)	
COPD, n (%)	1178 (7.4)	495 (5.8)	0.063	445 (5.5)	485 (6)	0.021
Systemic sepsis			0.058			0.015
No	15,136 (94.9)	8003 (94.3)		7615 (94.8)	7589 (94.5)	
SIRS	571 (3.6)	297 (3.5)		261 (3.3)	278 (3.5)	
Sepsis	196 (1.2)	166 (2)		134 (1.7)	140 (1.7)	
Septic shock	42 (0.3)	25 (0.3)		20 (0.2)	23 (0.3)	
Smoking, n (%)	2087 (13.1)	1267 (14.9)	0.053	1150 (14.3)	1200 (14.9)	0.018
DC, n (%)	1450 (9.1)	906 (10.7)	0.053	810 (10.1)	820 (10.2)	0.004
CHF, n (%)	297 (1.9)	122 (1.4)	0.033	125 (1.6)	118 (1.5)	0.007
Emergency, n (%)	969 (6.1)	582 (6.9)	0.032	510 (6.4)	537 (6.7)	0.014
Steroid, n (%)	503 (3.2)	224 (2.6)	0.031	187 (2.3)	221 (2.8)	0.027
BMI < 18.5	517 (3.2)	243 (2.9)	0.022	227 (2.8)	229 (2.9)	0.001
Dialysis, n (%)	128 (0.8)	52 (0.6)	0.023	54 (0.7)	52 (0.6)	0.003
Ventilator, n (%)	30 (0.2)	23 (0.3)	0.017	18 (0.2)	22 (0.3)	0.010
BWL, n (%)	1218 (7.6)	615 (7.2)	0.015	574 (7.1)	585 (7.3)	0.005
Ascites, n (%)	218 (1.4)	122 (1.4)	0.006	106 (1.3)	113 (1.4)	0.008
Renal failure, n (%)	46 (0.3)	22 (0.3)	0.006	25 (0.3)	21 (0.3)	0.009

ASA, American Society of Anesthesiologists; Wound II, clean/contamination; Wound III, contamination; Wound IV, dirty; COPD, chronic obstructive pulmonary disease; DC, disseminated cancer; CHF, congestive heart failure; BMI, body mass index; BWL, body weight loss.

**Table 2:** The association of postoperative 30-day mortality and morbidities in colon cancer patients divided by sidedness before propensity score matching.

Postop Outcomes	Full Cohort		OR (95% CI)	*p* Value
	Right Side (n = 15,945)	Left Side (n = 8491)		
Re-intubation	460 (2.9)	178 (2.1)	1.38 (1.16–1.63)	<0.001
Transfusion	2096 (13.1)	906 (10.7)	1.23 (1.15–1.33)	<0.001
UTI	524 (3.3)	222 (2.6)	1.26 (1.08–1.47)	0.004
DVT	294 (1.8)	121 (1.4)	1.29 (1.05–1.60)	0.016
30-day mortality	452 (2.8)	201 (2.4)	1.2 (1.02–1.41)	0.031
Organ SSI	512 (3.2)	303 (3.6)	0.9 (0.78–1.04)	0.138
PE	153 (1)	63 (0.7)	1.29 (0.97–1.73)	0.084
Septic shock	327 (2.1)	153 (1.8)	1.14 (0.94–1.38)	0.182
Pneumonia	482 (3)	237 (2.8)	1.08 (0.93–1.26)	0.307
Ventilator > 48 h	369 (2.3)	199 (2.3)	0.99 (0.83–1.17)	0.884
Return to OR	778 (4.9)	424 (5)	0.98 (0.87–1.10)	0.694
Superficial SSI	1019 (6.4)	552 (6.5)	0.98 (0.89–1.09)	0.738
Sepsis	570 (3.6)	318 (3.7)	0.96 (0.83–1.10)	0.498
Deep SSI	182 (1.1)	109 (1.3)	0.89 (0.70–1.13)	0.329
Wound disruption	178 (1.1)	106 (1.2)	0.89 (0.70–1.14)	0.359
MI	140 (0.9)	84 (1)	0.89 (0.68–1.16)	0.385
Stroke	74 (0.5)	45 (0.5)	0.88 (0.61–1.27)	0.481

Values in parentheses are percentages. UTI, urinary tract infection; DVT, deep vein thrombosis; SSI, surgical site infection; PE, pulmonary embolism; OR, operating room; MI, myocardial infarction.

**Table 3:** The association of postoperative 30-day mortality and morbidities in colon cancer patients divided by sidedness after propensity score matching.

Postop Outcomes	Matched Cohort (1:1)		OR (95% CI)	*p* Value
	Right Side (n = 8030)	Left Side (n = 8030)		
MI	57 (0.7)	83 (1)	0.69 (0.49–0.96)	0.027
Stroke	25 (0.3)	44 (0.5)	0.57 (0.35–0.93)	0.022
Transfusion	869 (10.8)	889 (11.1)	0.98 (0.90–1.07)	0.613
Ventilator > 48 h	171 (2.1)	190 (2.4)	0.90 (0.73–1.10)	0.312
Organ SSI	276 (3.4)	273 (3.4)	1.01 (0.86–1.19)	0.896
UTI	231 (2.9)	211 (2.6)	1.1 (0.91–1.35)	0.335
DVT	129 (1.6)	117 (1.5)	1.1 (0.86–1.41)	0.441
Deep SSI	95 (1.2)	106 (1.3)	0.90 (0.68–1.18)	0.435
30-day mortality	185 (2.3)	196 (2.4)	0.94 (0.77–1.15)	0.568
Superficial SSI	534 (6.7)	519 (6.5)	1.03 (0.92–1.16)	0.633
Pneumonia	209 (2.6)	230 (2.9)	0.91 (0.76–1.09)	0.310
Sepsis	292 (3.6)	297 (3.7)	0.98 (0.84–1.15)	0.834
Return to OR	398 (5)	401 (5)	0.99 (0.87–1.14)	0.913
Wound disruption	87 (1.1)	102 (1.3)	0.85 (0.64–1.13)	0.272
Re-intubation	179 (2.2)	173 (2.2)	1.04 (0.84–1.27)	0.746
Septic shock	155 (1.9)	146 (1.8)	1.06 (0.85–1.33)	0.6
PE	77 (1)	62 (0.8)	1.24 (0.90–1.73)	0.201

Values in parentheses are percentages. MI, myocardial infarction; SSI, surgical site infection; UTI, urinary tract infection; DVT, deep vein thrombosis; OR, operating room; PE, pulmonary embolism.

**Table 4:** Associated overall complications and total length of hospital stay in colon cancer patients divided by sidedness before and after propensity score matching.

Post-Operative Outcomes	Sidedness	Mean	B (Coefficient)	95% C.I.	*p* Value
Overall Complication ^b^	Right side	0.2053	0.017	0.004~0.029	0.011
	Left side	0.1886	0	0	
Length of total hospital stay ^b^	Right side	8.7372	0.327	0.114~0.539	0.003
	Left side	8.4104	0	0	
Overall Complication ^a^	Right side	0.1861	−0.005	−0.026~0	0.515
	Left side	0.1909	0	0	
Length of total hospital stay ^a^	Right side	8.1346	−0.362	−0.601~−0.123	0.003
	Left side	8.4962	0	0	

b: before; a: after.

## Data Availability

Data is contained within the article or Supplementary Materials.

## Supplementary Materials

The following supporting information can be downloaded at: https://www.mdpi.com/article/10.3390/jcm13133654/s1, Supplement S1: Revision (ICD-9) diagnosis codes and Current Procedural Terminology (CPT) codes. Supplement S2: 2013 ACS-NSQIP participant use data file.
