# Supplementary material for: Primary Tumor Sidedness Associated with Clinical Characteristics and Postoperative Outcomes in Colon Cancer Patients: A Propensity Score Matching Analysis
Source: J Clin Med. 2024 Jun 22;13(13):3654. doi: 10.3390/jcm13133654 (PMC11242415; doi:10.3390/jcm13133654)
Supplement: Supplementary file 1 [file jcm-13-03654-s001.zip › Supplement S1.pdf]

Supplement S1

ICD-9 code of colon cancer:

1. Right sided: 153.0, 153.1, 153.4, 153.6
2. Left sided: 153.2, 153.3, 153.7

CPT code for colectomy

1. 44140,44143,44144,44145,44146,44147,44160(RH)—partial colectomy, laparotomy
2. 44150,44151,44155,44156,44157,44158 →total colectomy, laparotomy
3. 44204,44205 (RH),44206,44207,44208→partial colectomy, laparoscopy
4. 44210,44211,44212→total colectomy, laparoscopy
