# Supplementary material for: Primary Tumor Sidedness Associated with Clinical Characteristics and Postoperative Outcomes in Colon Cancer Patients: A Propensity Score Matching Analysis
Source: J Clin Med. 2024 Jun 22;13(13):3654. doi: 10.3390/jcm13133654 (PMC11242415; doi:10.3390/jcm13133654)
Supplement: Supplementary file 1 [file jcm-13-03654-s001.zip › Supplement S2.pdf]

ACS NSQIP®

"Quality Improvement through Quality Data"

User Guide for the 2013 ACS NSQIP Participant Use Data File (PUF)

American College of Surgeons

National Surgical Quality Improvement Program

November 2014

American College of Surgeons

Inspiring Quality: Highest Standards, Better Outcomes

100+years

Contents

Section		Page
1.	Introduction	1
2.	Data Request Process	1
3.	File Description	2
4.	Data Collection Background and Data Quality	3
5.	Sampling Process and Case Inclusion/Exclusion Criteria	4
6.	Data Limitations	6
7.	Contact Information	7
8.	Frequently Asked Questions	7
9.	Data Variables and Definitions	11

1. Introduction

This document is designed to accompany the 2013 Participant Use Data File (PUF) available for download on the American College of Surgeons National Surgical Quality Improvement Program (ACS NSQIP) website (www.acsnsqip.org). The sections contained herein will provide the user with information on how to request the PUF, the contents of the data files, the data collection background, the inclusion and exclusion criteria for cases and hospitals, the data limitations, and the data point definitions and descriptions.

This user guide applies specifically to the 2013 PUF. Hospitals utilizing the PUF from a different year should refer to the user guide specifically tailored to that particular data set.

2. Data Request Process

An individual who has an official appointment at a fully enrolled site and wants to obtain a copy of the ACS NSQIP PUF can do so by visiting www.acsnsqip.org and following the steps listed below:

1. From the ACS NSQIP main page (www.acsnsqip.org) the requestor can scroll over "Program Specifics" as it appears on the banner. A drop down will appear, follow the drop down and put the mouse over "Quality Support Tools." As you are over "Quality Support Tools" you will see "Participant Use Data File" appear on the right, click on "Participant Use Data File."
2. Following a brief introduction, the requestor can click on "Request Data Set."
3. This will take the requestor to the Data Use Agreement. This is a 3-page document that implements the data protections of the Health Insurance Portability and Accountability Act of 1996 (HIPAA) and the ACS NSQIP Hospital Participation Agreement. Delivery of the PUF is contingent on agreement to the terms and conditions specified within the Data Use Agreement. You can read the Data Use Agreement from this page or download the 3-page document. The requestor is then required to type in their first and last name and click on "Request Data File." By clicking on "Request Data File" the requestor agrees to the terms and conditions of the Data Use Agreement.
4. Requestors will then be required to complete a brief online form to provide ACS with basic information about themselves, including the participating hospital in which they are currently employed and in what capacity, as well as how the requestor plans on using the PUF data. Once all of the required fields are completed, the requestor clicks "Submit."

5. ACS NSQIP staff will review the request in a timely manner. Program contacts at participating sites will be contacted at this time to confirm the requestor's affiliation with the hospital and confirm internal approval of the PUF request.
6. Following receipt and confirmation of the information submitted, an email will be sent to the requestor containing a username and password along with the URL to download the data. The web link will be active from the time of the email for 10 full days (240 hours).
7. The file will be available in 3 different formats (Text, SPSS, SAS) and depending on the connection speed should take between 5 and 30 minutes to download.
8. The requestor may be contacted to confirm receipt of the data file and allow for feedback on the delivery mechanism, data points contained, and data file format.

3. File Description

Each summer/fall a PUF will be made available for the previous calendar year's data. The PUF is available in 1 of 3 different formats - Text, SAS, and SPSS. In 2008, we provided an additional file that contains SAS and SPSS codes for constructing RACE variable that was available in previous years. The 2013 file contains 305 variables for each case, and a variable-by-variable description is provided starting on page 11.
A brief description of the different formats follows:

File Name	Type	Uncompressed File Size	Description
ACS_NSQIP_PUF13.txt	tab delimited TXT file	1.09GB	Contains 305 HIPAA compliant variables on 651,940 cases submitted from 435 sites in 2013.
ACS_NSQIP_PUF13.sas7bdat	SAS 9.2 data file	3.6 GB	Same information as stated above in TXT data format.
ACS_NSQIP_PUF13.sav<br><br>Construct_RACE_Codes.txt	SPSS 16.0 data file<br><br>Notepad file	4.1 GB<br><br>3KB	Same information as stated above in TXT and SAS data format. Contains SAS and SPSS codes for constructing RACE variable that was available in 2005, 2006 and 2007.

4. Data Collection Background and Data Quality

The ACS NSQIP collects data on over 300 variables, including preoperative risk factors, intraoperative variables, and 30-day postoperative mortality and morbidity outcomes for patients undergoing major surgical procedures in both the inpatient and outpatient setting. A site's trained and certified Surgical Clinical Reviewer (SCR) captures these data using a variety of methods including medical chart abstraction.

Required data variables are entered via web-based data collection to the ACS NSQIP website. Portions of the data may be automatically populated by a software program that was developed to extract data from the participating hospital's existing information systems. Requestors should contact the SCR(s) at their hospital for detailed information on how the hospital collects its ACS NSQIP data.

To ensure the data collected are of the highest quality, the ACS NSQIP has developed a host of different training mechanisms for the SCRs and conducts an Inter-Rater Reliability (IRR) Audit of selected participating sites. In addition to an initial web-based training program, the ACS NSQIP requires SCRs to complete a series of web-based training modules followed by a certification exam that must be retaken annually. The modules and certification exam focus on the program, processes, and analysis; preoperative, intraoperative, and postoperative definitions; and case studies. These modules are complemented by a growing online decision support system that ensures the SCRs have the knowledge and resources available to collect high-quality data.

The IRR Audit is a fundamental tool of ACS NSQIP to assess the quality of the data collected at participating sites. The process involves the review of multiple charts, some of which are selected randomly and others selected based on criteria designed to identify potential reporting errors. For example, cases with 5 or more preoperative risk factors and no reported mortality or morbidity or cases with 2 or fewer preoperative risk factors and reported mortality or morbidity will be selected for chart review. Operating room logs are also audited to ensure correct sampling of cases.

The combined results of the audits completed to date revealed an overall disagreement rate of approximately 2% for all assessed program variables. The ACS NSQIP has determined that an IRR Audit disagreement rate of 5% or less is acceptable. Sites that have higher than a 5% disagreement rate are not provided a hospital odds ratio in the ACS NSQIP Semiannual Report and may be required to undergo an additional audit following training and education recommendations from the ACS NSQIP.

5. Sampling Process and Case Inclusion/Exclusion Criteria

Sites participating in the ACS NSQIP can do so in a variety of options that cover general/vascular surgery, multispecialty surgery, or procedure targeted (reported separately). Each participation option includes a systematic sampling process that is described below.

Systematic Sampling Process

Many hospitals are not able to capture all of the surgical cases that meet the program's inclusion criteria. Therefore, a systematic sampling system called the 8-day cycle was developed to prevent bias in choosing cases for assessment. The SCR uses the 8-day cycle to select completed cases from the hospital's operative log. The schedule works as follows: If the first cycle begins on a Monday, it continues through to the following Monday (an 8-day period of time). The next cycle begins on Tuesday and continues through to the following Tuesday, and so on. There are 46 8-day cycles in 1 year, and the program requires that data be submitted for 42 of those cycles. The process ensures that cases have an equal chance of being selected from each day of the week. Case selection and case mix are monitored by the program on a weekly basis to ensure that the sampling is appropriate.

Case Inclusion Criteria

The following inclusion criteria were applied to cases collected in 2013. For the current inclusion/exclusion criteria please contact the ACS NSQIP Clinical Support Team at clinicalsupport@acsnsqip.org.

The ACS NSQIP includes all Major Cases. Major Cases are defined as:

- Cases performed under the following anesthesia types:
  - General
  - Spinal
  - Epidural
- The following cases *regardless* of anesthesia type:
  - Carotid endarterectomy
  - Inguinal herniorrhaphy
  - Parathyroidectomy
  - Thyroidectomy
  - Breast lumpectomy
  - Endovascular AAA repair

Case Exclusion Criteria

The following exclusion criteria were applied to cases collected in 2013. For the current inclusion/exclusion criteria please contact the ACS NSQIP Clinical Support Team at clinicalsupport@acsnsqip.org.

- Minor Cases (all cases that are not considered Major)
- Patients under the age of 18 years
- More than 3 inguinal herniorrhaphies in an 8-day period
- More than 3 breast lumpectomies in an 8-day period
- Trauma Cases - Specifically: A patient who is admitted to the hospital with acute trauma and has a surgical procedure(s) for that trauma will be excluded. Any operation performed after the patient has been discharged from the trauma stay will be included.
- Transplant Cases - Specifically: A patient who is admitted to the hospital for a transplant and has a transplant procedure and any additional surgical procedure during the transplant hospitalization will be excluded. Any operation performed after the patient has been discharged from the transplant stay will be included. ASA 6 (brain-dead organ donors)
- Concurrent Cases - An additional operative procedure performed by a different surgical team under the same anesthetic (for example, coronary artery bypass graft procedure on a patient who is also undergoing a carotid endarterectomy). An assessment is not required on the concurrent procedure; however, additional procedures would be repeated as “concurrent” in the operative section for the assessed case.
- Cases with CPT codes not on the CPT Code Inclusion List
- SCR on vacation - Each site is allowed to assign 4 of the 8-day cycles as vacation cycles and therefore does not need to collect cases during those cycles.

Hospital Exclusion Criteria

In addition to the case inclusion/exclusion criteria, hospital inclusion/exclusion criteria are also imposed. To maintain the highest level of data quality, only cases included in the odds ratio analysis are included in the PUF. These cases go through an additional level of scrutiny as they are passed from data collection to statistical analysis. A site is excluded from the odds ratio calculations and the PUF if it fits any of the following criteria:

- 30-day follow-up rate is under 80%
- Inter-Rater Reliability Audit disagreement rate is over 5%

Measures Sites Excluded

Due to the small number of “Measures” sites enrolled in ACS NSQIP, cases from “Measures” sites have been removed from the PUF dataset in order to protect against possible identification of those sites and cases.

6. Data Limitations

While every effort has been made to make the PUF as complete as possible, the data do have certain limitations. Some of these limitations have been deliberately introduced to safeguard the privacy of patients (such as removal of absolute dates). Other limitations are due to resource constraints (such as the collection of generic surgical variables only, except for the procedure targeted option, which is reported separately). The following items represent the most salient limitations of the data:

- Because such a wide variety of operations are tracked, the variables are necessarily generic in nature. This limitation may pose difficulties for researchers attempting in-depth research on specific conditions or operations.

- While the sex and race distributions are reasonably representative of the national surgery patient population, only patients over the age of 18 are available for assessment, so the age distribution is somewhat truncated. Patients over the age of 90 are also grouped into a 90+ category to prevent cases from being identifiable due to unique data.

- Patients are followed after surgery for 30 days. Complications or death after that period are not included. Hospitals may follow patients longer than 30 days, but this data is not reported by NSQIP.

- In order to comply with HIPAA requirements, all absolute dates have been removed. The most critical of these is the date of surgery, which has been reduced to year of surgery only. Some dates (hospital entry, dates of laboratory tests, and so on) have been recoded into durations e.g. Date of Admission and Date of Discharge is recoded into Hospital Length of Stay.

- In order to comply with the Hospital Participation Agreement (HPA) that is agreed to between the ACS and participating sites, facility identifiers as well as geographic information regarding the case have been removed. The HPA stipulates that the ACS does not identify participating sites. Site identification

could be possible even with blinded identifiers through advanced statistics. A stipulation of access to the PUF is completion of the Data Use Agreement that strictly prohibits attempts to identify hospitals, health care providers, or patients.

- While many risk factors are tracked, preventative measures are not recorded which can lead to an underestimation of the risk of certain conditions when such measures are routinely taken before surgery.

- The data are submitted from hospitals that are participating in the ACS NSQIP and do not represent a statistically valid nationally representative sample.

- Most patients do not receive all possible preoperative laboratory tests, so some of these variables have a high percentage of missing values (15% to 45%, depending on the tests). This high percentage of missing data can make it problematic to use these variables in a traditional logistic regression model as well as in many other types of analysis.

This list may not include all data limitations and additional limitations may apply in future versions of the data.

7. Contact Information

All questions about the User Guide or PUF, as well as comments and suggestions for improvements are welcome and may be directed to Brian Matel, ACS NSQIP Statistical Report Manager, via email at bmatel@facs.org.

8. Frequently Asked Questions

Request Process

Q: Who has access to this file?
A: Any individual with an official appointment at a fully participating site will be given access to the file following completion of the Data Use Agreement and a short set of questions that are available on the website.

Q: Is the file available to individuals from nonparticipating sites?
A: At this time the data files are only available to individuals with official appointments at fully participating sites.

Q: I am at a participating site and would like to work on a research project with others from a different site that is not participating. Will I be allowed to do that?
A: No. At this time use of the file is restricted to individuals at fully participating sites.

Q: How do I obtain a copy of this file?
A: Please see the "Data Request Process" on page 1 of this document for a step-by-step approach on how to do so.

Contents of the Files

Q: What is in this file?
A: The file contains Health Insurance Portability and Accountability Act (HIPAA) de-identified data from sites participating in the ACS NSQIP that received odds ratios in 2013. Each record includes 305 variables. The variable name, variable label, data definition, and other pertinent information are provided in Section 10: Data Variables and Definitions.

Q: Are other PUF data sets available?
A: Six other PUF files are available for download:
2005/2006 PUF – 152,490 cases from 121 sites
2007 PUF – 211,407 cases from 183 sites
2008 PUF – 271,368 cases from 211 sites
2009 PUF – 336,190 cases from 237 sites
2010 PUF – 363,431 cases from 258 sites
2011 PUF – 442,149 cases from 315 sites
2012 PUF – 543,885 cases from 374 sites

Q: Are site identifiers included in the database?
A: At this time we do not provide any geographic or site-specific identification. We took this approach to ensure the privacy of both the participating sites and surgeons.

Q: Are there surgeon-specific identifiers included in the database?
A: At this time we do not provide any surgeon-specific information. We took this approach to ensure the privacy of both the participating sites and surgeons.

Q: Why does the PUF exclude specific dates?

A: In order to release the PUF, certain adjustments to the data are required to ensure proper protection of patient information. To meet these requirements, we remove all elements of dates (except quarter of admission and year) for dates directly related to an individual. For more information on the 18 data elements that are required for removal, please visit http://privacyruleandresearch.nih.gov/ or http://privacyruleandresearch.nih.gov/pdf/HIPAA_Booklet_4-14-2003.pdf.

Q: The ACS NSQIP program collects over 150 variables, but the database contains 305 variables. What are the additional variables?

A: The additional variables contained in the PUF relate to computed durations. For example, the admission and discharge dates are used to calculate hospital length of stay. In addition, each complication in the ACS NSQIP requires the use of 3 different variables in the database. There are a few other data elements collected in the ACS NSQIP that require multiple variables in the database. In 2008, we've removed RACE variable but added RACE_NEW and ETHNICITY_HISPANIC variables to comply with the CMS standard.

Q: I am the Surgeon Champion or Surgical Clinical Reviewer from a site that has records in the PUF and would like to know which specific records are ours.

A: You may contact Brett Beemer, ACS NSQIP Application Support Specialist, via email at bbeemer@facs.org to request a file that will contain the Case IDs from your facility.

Values in the Data

Q: For each of the following complications, Pneumonia, On Ventilator > 48 hours, Urinary Tract Infection, and Bleeding Transfusion, one case did not have a known duration from operation to complication. Why is that?

A: In each of these complications the case had an invalid date which inhibited the calculation of duration. The number of days from operation to complication variable is coded as -99 for these cases.

Q: What are the probability scores for mortality and morbidity and how often are they calculated?

A: The probabilities of mortality and morbidity are provided in this database for all surgery cases in 2013. These probabilities are derived using hierarchical regression analysis, but based only on patient-level effects. They represent the probability (0 to1) that a case will experience a morbid or mortal event based on the pre-existing conditions. These probabilities are calculated every 6 months for

the previous 12 months of data so the algorithm used to generate the predicted values changes over time as does the data used to create the algorithm.

Q: Which calculated probabilities of mortality and morbidity are supplied in this data set?

A: The probabilities of mortality and morbidity for all surgical cases used in the risk-adjusted analysis in 2013 are provided. Future versions of the PUF may contain a more complete set of predictive values.

Q: Why do some of the preoperative lab values have duration from lab to operation, but a value of -99 for the lab value?

A: The results of the lab tests can be entered manually and thus are susceptible to data entry error. Depending on the preoperative lab variable roughly 1% of the cases had invalid values and these invalid values were set to -99 to simplify analysis. It is also possible that some cases have valid lab values, but are missing duration from lab to operation variable. This discrepancy is also related to a data entry error and the program continues to improve the data collection software to minimize the potential for data entry errors.

Q: When performing analysis on the five digit CPT codes in the Other and Concurrent variables, how should I interpret those cases with a valid 5 digit CPT code but a CPT description set to NULL?

A: If the case has a valid 5 digit CPT code that procedure occurred and should be evaluated as such. The CPT description is a secondary variable and provided for convenience. In the processing of large amounts of data some descriptions are purposefully or inadvertently removed.

File Formats

Q: In what file formats are the data available?

A: The data files are made available in a tab delimited TXT file, an SPSS file, and a SAS file.

*** When a change in definitions across PUF years is noted, users should attend to this if they merge files. It is suggested that they evaluate variable categories across years and combine them in a manner appropriate to their research objectives.**

VARIABLE ADDED IN 2011
VARIABLE ADDED IN 2012
VARIABLE ADDED IN 2013

Position #	Variable Name	Data Type	Variable Label	Variable Definition	Variable Options at Entry	Comments
1	CaseID	Num	Case Identification Number	Each case or record in the database has a unique CaseID number.		
2	SEX	Char	Gender	Gender	Male; Female	NULL = Unknown
3	RACE_NEW	Char	New Race	Race	American Indian or Alaska Native<br>Asian<br>Black or African American<br>Native Hawaiian or Pacific Islander<br>Unknown/Not Reported<br>White	NULL = No Response
4	ETHNICITY_HISPANIC	Char	Ethnicity Hispanic	Ethnicity Hispanic	Yes; No; Unknown	NULL = No Response
5	PRNCPTX	Char	Principal operative procedure CPT code description	The principal operative procedure is the most complex of all the procedures performed by the primary operating team during the trip to the operating room. Additional procedures requiring separate CPT codes and/or concurrent procedures will be entered separately in the "Other Procedures" or "Concurrent Procedures" categories.		
6	CPT	Char	CPT	The CPT code of the principal operative procedure.		
7	WORKRVU	Num	Work Relative Value Unit	Work Relative Value Unit		-99 = Unknown
8	INOUT	Char	Inpatient/outpatient	The hospital's definition of inpatient and outpatient status.	Outpatient; Inpatient	NULL = Unknown
9	TRANST	Char	Transfer status	The patient's transfer status which includes the following options: Admitted directly from home (Includes patients arriving from another hospital's emergency department); If the patient was transferred from another facility and was considered an inpatient at that facility Acute Care Hospital, VA Acute Care Hospital, Chronic Care Facility, and VA Chronic Care Facility are acceptable. If the kind of facility could not be determined 'Other' is entered.	From acute care hospital inpatient<br>Not transferred (admitted from home)<br>Nursing home - Chronic care - Intermediate care<br>Outside emergency department<br>Transfer from other<br>Unknown	NULL = No Response<br>Definition change from 2009
10	Age	Char	Age of patient with patients over 89 coded as 90+	Age of patient with patients over 89 coded as 90+. No patients under 15 are included.		-99 = Unknown
11	AdmYR	Num	Year of Admission	Year of admission to the hospital		-99 = Unknown
12	AdmSYR	Num	Year of Admission to Surgery	Year of admission to the surgical service		Historical variable, no longer used
13	OperYR	Num	Year of Operation	Year the surgical procedure is performed		-99 = Unknown
14	DISCHDEST	Char	Discharge Destination	Designate whether the patient was discharged to home or to another type of facility. Choose the patient's discharge destination from the following selections:<br>(1) Skilled care, not home (e.g., transitional care unit, subacute hospital, ventilator bed, skilled nursing home)<br>(2) Unskilled facility, not home (e.g., nursing home or assisted facility-if not patient's home preoperatively)<br>(3) Facility which was home (e.g., return to a chronic care, unskilled facility, or assisted living-which was the patient's home preoperatively)<br>(4) Home<br>(5) Separate acute care (e.g., transfer to another acute care facility)<br>(6) Rehab<br>(7) Expired<br>(8) Unknown	Skilled Care, Not Home<br>Unskilled Facility Not Home<br>Facility Which was Home<br>Home<br>Separate Acute Care<br>Rehab<br>Expired<br>Unknown	NULL = No Response Variable added in 2011
15	ANESTHES	Char	Principal anesthesia technique	The principal anesthesia technique used. General anesthesia takes precedence over all other forms of anesthesia. If the patient is given a regional/spinal or epidural and MAC, MAC anesthesia would take precedence.	Epidural<br>General<br>Local<br>Monitored Anesthesia care (MAC) / IV Sedation<br>None<br>Other<br>Regional<br>Spinal<br>Unknown	NULL = No Response Definition revised or clarified from 2011

Position #	Variable Name	Data Type	Variable Label	Variable Definition	Variable Options at Entry	Comments
16	ATTEND	Char	Level of Residency Supervision	Highest level of supervision provided by the attending staff surgeon for the case. Attending alone: Staff practitioner performed the procedure; resident not present; Attending in OR: Staff practitioner is scrubbed and present in the procedure/operating room;	Attending & Resident in OR	NULL = Unknown<br>Definition change from 2009
				Attending in OR Suite: Staff practitioner is present in the procedural/surgical suite and available for consultation; Attending Not Present, but Available: Staff practitioner is not present, but immediately available on campus.	Attending Alone<br>Attending Not Present, but Available	
17	SURGSPEC	Char	Surgical Specialty	The surgical specialty area that best characterizes the principal operative procedure.<br>• Surgeon's self-declared specialty<br>• If a surgeon is privileged to perform cases within multiple specialties (regardless of board certification), the service line/specialty most closely related to the principal operative procedure would be assigned.<br>• If a Surgeon is "Board Certified" in both Vascular Surgery and General Surgery and performs an appendectomy, the surgeon's specialty should be designated as general surgery, but if he/she performs a vascular bypass, it should be designated as vascular.	Cardiac Surgery<br>General Surgery<br>Gynecology<br>Neurosurgery<br>Orthopedics<br>Otolaryngology (ENT)<br>Plastics<br>Thoracic<br>Urology<br>Vascular<br>Unknown<br>Other<br>Interventional Radiologist	Definition revised or clarified from 2013
18	ELECTSURG	Char	Elective Surgery	"YES" is entered if the patient is brought to the hospital or facility for a scheduled (elective) surgery from their home or normal living situation on the day that the procedure is performed.<br><br>ENTER NO (Exclude) FOR the following:<br>● patients who are inpatient at an acute care hospital (example: patient transferred<br>from another acute care hospital to your hospital for surgery)<br>● patients who are transferred from an ED<br>● patients who are transferred from a clinic<br>● patients who undergo an emergent/urgent surgical case<br>● patients admitted to the hospital on the day(s) prior to a scheduled procedure for any reason (e.g. cardiac or pulmonary workup or "tuning", bowel cleanout, TPN, hydration, anticoagulation reversal etc.)<br><br>ENTER YES (Include) FOR the following:<br>● patients staying with friends or family, or in a local hotel, because of logistics (example: patient lives 50 miles from the hospital and stays in a hotel across from the hospital the night before their scheduled (elective) surgery)<br>● patients who come from their present "home" (which may include patients whose home is a nursing home, assisted care facility, prison or other non-hospital institution)<br><br>*The intent is to identify a relatively homogeneous group of patients who are well enough to come from home, to allow for more meaningful comparative analyses.*	Yes; No; Unknown	NULL = No Response Variable added in 2011
19	HEIGHT	Num	Height	The patient's most recent height documented in the medical record in either inches (in) or centimeters (cm), within the 30 days prior to the principal operative procedure or at the time the patient is being considered a candidate for surgery		-99 = Unknown<br>Definition revised in 2013
20	WEIGHT	Num	Weight	The patient's most recent weight documented in the medical record in either pounds (lbs.) or kilograms (kg), within the 30 days prior to the principal operative procedure or at the time the patient is being considered a candidate for surgery		-99 = Unknown<br>Definition revised in 2013

Position #	Variable Name	Data Type	Variable Label	Variable Definition	Variable Options at Entry	Comments
21	DIABETES	Char	Diabetes mellitus with oral agents or insulin	The treatment regimen of the patient's chronic, long-term management (> 2 weeks). Diabetes mellitus is a metabolic disorder of the pancreas whereby the individual requires daily dosages of exogenous parenteral insulin or a non-insulin anti-diabetic agent to prevent a hyperglycemia/metabolic acidosis. Patients with insulin resistance that routinely take anti-diabetic agents are included. Patients whose diabetes is controlled by diet alone are not included. No: no diagnosis of diabetes or diabetes controlled by diet alone. Non-Insulin: a diagnosis of diabetes requiring therapy with a non-insulin anti-diabetic agent (such as oral agents or other non-insulin agents). Insulin: a diagnosis of diabetes requiring daily insulin therapy	INSULIN; NO; NON-INSULIN	NULL = Unknown<br>Definition change from 2009<br>Definition revised or clarified from 2010
22	SMOKE	Char	Current smoker within one year	If the patient has smoked cigarettes in the year prior to admission for surgery "YES" entered. Patients who smoke cigars or pipes or use chewing tobacco are not included.	Yes; No	NULL = Unknown
23	PACKS	Num	Pack-years of smoking	If the patient has ever been a smoker, the total number of pack/years of smoking for this patient is provided. Pack-years are defined as the number of packs of cigarettes smoked per day times the number of years the patient has smoked. If the patient has never been a smoker, "0" is entered. If pack-years are > 200, 200 is entered. If smoking history cannot be determined, "-99" is entered. The possible range for number of pack-years is 0 to 200. If the chart documents differing values for pack year cigarette history or ranges for either packs per day or number of years patient has smoked, the highest value is documented.		-99 = Unknown
24	ETOH	Char	EtOH > 2 drinks/day in 2 wks before admission	"YES" is entered if 2 drinks per day in the two weeks prior to admission: The patient admits to drinking >2 ounces of hard liquor or > two 12 oz. cans of beer or > two 6 oz. glasses of wine per day in the two weeks prior to admission. If the patient is a binge drinker, the numbers of drinks during the binge are divided by seven days and then the definition is applied.	Yes; No	NULL = Unknown
25	DYSPNEA	Char	Dyspnea	Dyspnea may be symptomatic of numerous disorders that interfere with adequate ventilation or perfusion of the blood with oxygen and is defined as difficult, painful or labored breathing. The intent of this variable is to capture the usual or typical level of dyspnea (patient's baseline), within the 30-days prior to surgery. The intent is not to include patients solely because of an acute respiratory condition leading to intubation prior to surgery, but rather to reflect a chronic disease state. Characterize the patient's dyspnea status when they were in their usual state of health, prior to the onset of the acute illness, within the 30 days prior to surgery.<br>(1) No dyspnea<br>(2) Dyspnea upon moderate exertion (for example-is unable to climb one flight of stairs without shortness of breath) (3) Dyspnea at rest (for example: cannot complete a sentence without needing to take a breath)<br>Note: Acute pre-op dyspnea associated with the acute illness will be captured through other variables like pre-op vent dependence, emergency status or ASA Class. The previous requirement that the patient has to themselves state that they are symptomatic has been removed: not all patients are able to verbalize this symptomatology.	AT REST; MODERATE EXERTION; No	NULL = Unknown Definition revised or clarified from 2011
26	DNR	Char	Do not resuscitate (DNR) status	If the patient has had a Do-Not-Resuscitate (DNR) order written in the physician's order sheet of the patient's chart and it has been signed or co-signed by an attending physician, enter "YES". There must be active DNR order at the time the patient is going to the OR. However, if the DNR order, as defined above, was rescinded immediately prior to surgery, in order to operate on the patient, enter "YES". Answer "NO" if DNR discussions are documented in the progress note, but no official DNR order has been written in the physician order sheet or if the attending physician has not signed the official order. Also answer "NO" if the patient is admitted as a DNR from a nursing home, as there must be a new DNR order written and signed/co-signed by a hospital attending physician. Advanced Directives are not DNR orders.	Yes; No	NULL = Unknown Definition revised or clarified from 2011
27	FNSTATUS1	Char	Functional health status Prior to Current Illness		Independent	Historical variable, no longer used

Position #	Variable Name	Data Type	Variable Label	Variable Definition	Variable Options at Entry	Comments
28	FNSTATUS2	Char	Functional health status Prior to Surgery	This variable focuses on the patient's abilities to perform activities of daily living (ADLs) in the 30 days prior to surgery. Activities of daily living are defined as 'the activities usually performed in the course of a normal day in a person's life'. ADLs include: bathing, feeding, dressing, toileting, and mobility. The <u>best</u> functional status demonstrated by the patient within the 30 days prior to surgery is reported. Report the level of functional health status as defined by the following criteria.<br>(1) Independent: The patient does not require assistance from another person for any activities of daily living. This includes a person who is able to function independently with prosthetics, equipment, or devices.<br>(2) Partially dependent: The patient requires some assistance from another person for activities of daily living. This includes a person who utilizes prosthetics, equipment, or devices but still requires some assistance from another person for ADLs.<br>(3) Totally dependent: The patient requires total assistance for all activities of daily living.<br>(4) Unknown: If unable to ascertain the functional status prior to surgery, report as unknown.<br><br>All patients with psychiatric illnesses should be evaluated for their ability to function with or without assistance with ADLs just as the non-psychiatric patient. For instance, if a patient with schizophrenia is able to care for him/herself without the assistance of nursing care, he/she is considered independent. If there is a change in the patients functional status, (i.e. improvement to worsening) within the 30 days prior to surgery, report the patient's best functional status.	Independent; Partially Dependent; Totally Dependent; Unknown	NULL = No Response Definition revised or clarified from 2010
29	VENTILAT	Char	Ventilator dependent	"YES" is entered if a preoperative patient required ventilator-assisted respiration at any time during the 48 hours preceding surgery. This does not include the treatment of sleep apnea with CPAP.	Yes; No	NULL = Unknown
30	HXCOPD	Char	History of severe COPD	COPD [emphysema and/or chronic bronchitis/bronchiectasis/ bronchiolitis obliterans organizing pneumonia (BOOP)] is a progressive disease that makes it hard to breathe. 'Progressive' means the disease gets worse over time. "COPD can cause coughing that produces large amounts of mucus . . ., wheezing, shortness of breath, chest tightness, and other symptoms" (National Heart Lung and Blood Institute, 2010) 2.<br>Medical record must document that there is a historical or current diagnosis of COPD AND at least one of the following, within the 30 days prior to the principal operative procedure or at the time the patient is being considered as a candidate for surgery:<br>Functional disability from COPD (e.g., dyspnea, inability to perform ADLs)<br>Or Requires chronic bronchodilator therapy with oral or inhaled agents<br>Or Hospitalization in the past for treatment of COPD<br>Or An FEV1 of <75% of predicted on a prior pulmonary function test (PFT)	Yes; No	NULL = Unknown<br>Definition revised or clarified from July 2013
31	CPNEUMON	Char	Current pneumonia	"YES" is entered if the patient has a new pneumonia or recently diagnosed pneumonia and on current antibiotic treatment at the time the patient is brought to the OR. Patients with pneumonia *must meet criteria from both <u>Radiology</u> and <u>Signs/Symptoms/Laboratory</u> sections* listed as follows:<br><br><u>Radiology:</u><br>One definitive chest radiological exam (x-ray or CT)* with at least <u>one</u> of the following:<br>•New or progressive and persistent infiltrate<br>•Consolidation or opacity<br>•Cavitation<br>Note: In patients with underlying pulmonary or cardiac disease (e.g. respiratory distress syndrome, bronchopulmonary dysplasia, pulmonary edema, or chronic obstructive pulmonary disease), <u>two or more serial chest radiological exams (x-ray or CT)</u> are required. (Serial radiological exams should be taken no less than 12 hours apart, but not more than 7 days apart. The occurrence should be assigned on the date the patient first met all of the criteria of the definition (i.e, if the patient meets all PNA criteria on the day of the first xray, assign this date to the occurrence. Do not assign the date of the occurrence to when the second serial xray was performed).	Yes; No	Definition revised or clarified from 2010<br>NULL=Unknown

Position #	Variable Name	Data Type	Variable Label	Variable Definition	Variable Options at Entry	Comments
	CPNEUMON (Continued)	Char	Current pneumonia	Signs/Symptoms/Laboratory:<br>FOR ANY PATIENT, at least one of the following:<br>•Fever (>38 C or >100.4 F) with no other recognized cause<br>•Leukopenia (<4000 WBC/mm3) or leukocytosis(≥12,000 WBC/mm3)<br>•For adults ≥ 70 years old, altered mental status with no other recognized cause<br>And<br>At least one of the following:<br>•5% Bronchoalveolar lavage (BAL) -obtained cells contain intracellular bacteria on direct microscopic exam (e.g., Gram stain)<br>•Positive growth in blood culture not related to another source of infection<br>•Positive growth in culture of pleural fluid<br>•Positive quantitative culture from minimally contaminated lower respiratory tract (LRT) specimen (e.g. BAL or protected specimen brushing)<br>OR<br>At least two of the following:<br>•New onset of purulent sputum, or change in character of sputum, or increased respiratory secretions, or increased suctioning requirements<br>•New onset or worsening cough, or dyspnea, or tachypnea<br>•Rales or rhonchi<br>•Worsening gas exchange (e.g. O2 desaturations (e.g., PaO2/FiO2 ≤ 240), increased oxygen requirements, or increased ventilator demand)		
32	ASCITES	Char	Ascites	"YES" is entered for patients with the presence of fluid accumulation in the peritoneal cavity noted on physical examination, abdominal ultrasound, or abdominal CT/MRI within 30 days prior to the operation. Documentation should state either active or a history of liver disease (for example, jaundice, encephalopathy, hepatomegaly, portal hypertension, liver failure, or spider telangiectasia). Minimal or trace ascites would not qualify; however; malignant ascites (exclusive of liver disease) due to extensive cancer would qualify.	Yes; No	Definition revised or clarified from 2010<br>NULL=Unknown
33	ESOVAR	Char	Esophageal varices	"YES" is entered for patients with esophageal varices present preoperatively and documented on an EGD or CT scan performed within 6 months prior to the surgical procedure. Esophageal varices are engorged collateral veins in the esophagus that bypass a scarred liver to carry portal blood to the superior vena cava. A sustained increase in portal pressure results in esophageal varices that are most frequently demonstrated by direct visualization at esophagoscopy.	Yes; No	NULL = Unknown
34	HXCHF	Char	Congestive heart failure (CHF) in 30 days before surgery	"YES" is entered in patients with congestive heart failure. Congestive heart failure is the inability of the heart to pump a sufficient quantity of blood to meet the metabolic needs of the body or can do so only at increased ventricular filling pressure. Only newly diagnosed CHF within the previous 30 days or a diagnosis of chronic CHF with new signs or symptoms in the 30 days prior to surgery fulfills this definition. Common manifestations are: -Abnormal limitation in exercise tolerance due to dyspnea or fatigue -Orthopnea (dyspnea on lying supine) -Paroxysmal nocturnal dyspnea (PND-awakening from sleep with dyspnea) -Increased jugular venous pressure -Pulmonary rales on physical examination -Cardiomegaly -Pulmonary vascular engorgement.	Yes; No	NULL = Unknown
35	HXMI	Char	History of myocardial infarction 6 mos prior to surgery	"Yes" is entered for patients with a history of a non-Q wave or a Q wave infarct in the six months prior to surgery as diagnosed in the patient's medical record.	Yes; No	NULL = Unknown
36	PRVPCI	Char	Previous PCI	"YES" is entered for patient who have undergone percutaneous coronary intervention (PCI) at any time (including any attempted PCI). This includes either balloon dilatation or stent placement. This does not include valvuloplasty procedures.	Yes; No	NULL = Unknown
37	PRVPCS	Char	Previous cardiac surgery	"YES" is entered if the patient has had any major cardiac surgical procedures (performed either as an 'off-pump' repair or utilizing cardiopulmonary bypass). This includes coronary artery bypass graft surgery, valve replacement or repair, repair of atrial or ventricular septal defects, great thoracic vessel repair, cardiac transplant, left ventricular aneurysmectomy, insertion of left ventricular assist devices (LVAD), etc. Not include are pacemaker insertions or automatic implantable cardioverter defibrillator (AICD) insertions.	Yes; No	NULL = Unknown

Position #	Variable Name	Data Type	Variable Label	Variable Definition	Variable Options at Entry	Comments
38	HXANGINA	Char	History of angina in 1 month before surgery	"YES" is entered if patient reports pain or discomfort between the diaphragm and the mandible resulting from myocardial ischemia. Typically angina is a dull, diffuse (fist-sized or larger) substernal chest discomfort precipitated by exertion or emotion and relieved by rest or nitroglycerine. Radiation to the arms and shoulders often occurs, and occasionally to the neck, jaw (mandible, not maxilla), or interscapular region. For patients on anti-anginal medications, 'YES' is entered only if the patient has had angina at any time within one month prior to surgery.	Yes; No	NULL = Unknown
39	HYPERMED	Char	Hypertension requiring medication	Hypertension (HTN) is the term used to describe high blood pressure. Blood pressure is a measurement of the force against the walls of your arteries as your heart pumps blood through your body. High blood pressure (hypertension) is when your blood pressure is 140/90 mmHg or above most of the time."<br>The diagnosis of HTN must be documented in the patient's medical record and the condition is severe enough that it requires antihypertensive medication, within 30 days prior to the principal operative procedure or at the time the patient is being considered as a candidate for surgery. The patient must have been receiving or required long-term treatment of their chronic hypertension for > 2 weeks.	Yes; No	NULL = Unknown Definition revised or clarified from July 2013
40	HXPVD	Char	History of revascularization/amputation for periph. vascular disease	"YES" is entered for a patient with any type of angioplasty (including stent placement) or revascularization procedure for atherosclerotic peripheral vascular disease (PVD) (e.g., aorta-femoral, femoral-femoral, femoral-popliteal) or a patient who has had any type of amputation procedure for PVD (e.g., toe amputations, transmetatarsal amputations, below the knee or above the knee amputations). Patients who have had amputation for trauma or a resection of abdominal aortic aneurysms should not be included.	Yes; No	NULL = Unknown
41	RESTPAIN	Char	Rest pain/gangrene	"YES" is entered for a patient with rest pain or Gangrene. Rest pain is a more severe form of ischemic pain due to occlusive disease, which occurs at rest and is manifested as a severe, unrelenting pain aggravated by elevation and often preventing sleep. Gangrene is a marked skin discoloration and disruption indicative of death and decay of tissues in the extremities due to severe and prolonged ischemia. Patients included with ischemic ulceration and/or tissue loss related to peripheral vascular disease. Fournier's gangrene are not included.	Yes; No	NULL = Unknown
42	RENAFAIL	Char	Acute renal failure (pre-op)	A clinical condition associated with rapid decline of kidney function. The intent of this variable is to capture the situation where the patient's renal function has demonstrated compromise within 24 hours prior to surgery.<br>Patient must meet ONE of the following scenarios (A or B) within 24 hours prior to the principal operative procedure:<br>A. An increase in BUN based on two measurements and two creatinine (Cr) results above 3mg/dl. There must be at minimum two measurements per lab value, the most recent of which must be within 24 hours prior to the start of the principal operative procedure; the second must be within 90 days of the principal operative procedure.<br>B. The surgeon or attending physician has documented Acute Renal Failure in the medical record and the patient demonstrates ONE of the following:<br>1) An increase in BUN based on at least two measurements, the most recent of which must be within 24 hours prior to the start of the principal operative procedure; the second must be within 90 days of the principal operative procedure and one creatinine above 3mg/dl, which must be within 24 hours prior to the start of the principal operative procedure.<br>2) Two creatinine results above 3mg/dl, the most recent of which must be within 24 hours prior to the start of the principal operative procedure; the second must be within 90 days of the principal operative procedure and one abnormal BUN (based on your hospital's reference range for BUN), which must be within 24 hours prior to the start of the principal operative procedure.	Yes; No	NULL = Unknown Definition revised or clarified from 2013
43	DIALYSIS	Char	Currently on dialysis (pre-op)	"YES" is entered if the patient has acute or chronic renal failure requiring treatment with peritoneal dialysis, hemodialysis, hemofiltration, hemodiafiltration, or ultrafiltration within 2 weeks prior to the principal operative procedure. The medical record must document that such a treat was indicated.	Yes; No	NULL = Unknown

Position #	Variable Name	Data Type	Variable Label	Variable Definition	Variable Options at Entry	Comments
44	IMPSENS	Char	Impaired sensorium	"YES" is entered if patient is acutely confused and/or delirious and responds to verbal and/or mild tactile stimulation. Patients is noted to have developed an impaired sensorium if they have mental status changes, and/or delirium in the context of the current illness. Patients with chronic or long-standing mental status changes secondary to chronic mental illness (e.g., schizophrenia) or chronic dementing illnesses (e.g., multi-infarct dementia, senile dementia of the Alzheimer's type) are not included. This assessment of the patient's mental status is within 48 hours prior to the surgical procedure. Example: A patient is admitted to the orthopedics service after a fall with a fractured hip. The patient is also noted to be dehydrated and febrile. He is disoriented to place and time and seems confused. His family reports that he has been oriented and alert prior to the fall. This patient has an impaired sensorium on the basis of his confusion and disorientation.	Yes; No	NULL = Unknown
45	COMA	Char	Coma >24 hours	"YES" is entered if patient is unconscious, or postures to painful stimuli, or is unresponsive to all stimuli entering surgery. This does not include drug-induced coma.	Yes; No	NULL = Unknown
46	HEMI	Char	Hemiplegia	"YES" is entered if patient has sustained acute or chronic neuromuscular injury resulting in total or partial paralysis or paresis (weakness) of one side of the body. 'YES' is entered if the patient has hemiplegia/hemiparesis (that has not recovered or been rehabilitated) upon arrival to the OR. "YES" is entered, if there is hemiplegia or hemiparesis associated with a CVA/Stroke also.	Yes; No	NULL = Unknown
47	HXTIA	Char	History of transient ischemic attacks (TIA)	"YES" is entered if patient has transient ischemic attacks (TIAs). TIAs are focal neurologic deficits (e.g. numbness of an arm or amaurosis fugax) of sudden onset and brief duration (usually <30 minutes) that usually reflects dysfunction in a cerebral vascular distribution. These attacks may be recurrent and, at times, may precede a stroke.	Yes; No	NULL = Unknown
48	CVA	Char	CVA/Stroke with neurological deficit	"YES" is entered if patient has a history of a cerebrovascular accident (embolic, thrombotic, or hemorrhagic) with persistent residual motor, sensory, or cognitive dysfunction. (e.g., hemiplegia, hemiparesis, aphasia, sensory deficit, impaired memory). If the neurological deficit is hemiplegia/hemiparesis, 'YES' is entered to Hemiplegia/Hemiparesis in addition to CVA/Stroke.	Yes; No	NULL = Unknown
49	CVANO	Char	CVA/Stroke with no neurological deficit	"YES" is entered if the patient has a history of a cerebrovascular accident (embolic, thrombotic, or hemorrhagic), but no current residual neurologic dysfunction or deficit.	Yes; No	NULL = Unknown Definition revised or clarified from 2010
50	TUMORCNS	Char	Tumor involving CNS	"YES" is entered if patient has a space-occupying tumor of the brain or spinal cord, which may be benign (e.g., meningiomas, ependymoma, oligodendroglioma) or primary (e.g., astrocytoma, glioma, glioblastoma multiform) or secondary malignancies (e.g., metastatic lung, breast, malignant melanoma). Other tumors that may involve the CNS include lymphomas and sarcomas. "YES" is entered even if the tumor was not treated.	Yes; No	NULL = Unknown
51	Para	Char	Paraplegia	"YES" is entered if the patient has sustained acute or chronic neuromuscular injury resulting in total or partial paralysis or paresis (weakness) of the lower extremities.	Yes; No	NULL = Unknown
52	QUAD	Char	Quadriplegia	"YES" is entered if the patient has sustained acute or chronic neuromuscular injury resulting in total or partial paralysis or paresis (weakness) of all four extremities.	Yes; No	NULL = Unknown

Position #	Variable Name	Data Type	Variable Label	Variable Definition	Variable Options at Entry	Comments
53	DISCANCR	Char	Disseminated cancer	"YES" is entered for patients who have cancer that: (1) Has spread to one site or more sites in addition to the primary site AND (2) In whom the presence of multiple metastases indicates the cancer is widespread, fulminant, or near terminal. The following are reported as Disseminated Cancer: Acute Lymphocytic Leukemia (ALL), Acute Myelogenous Leukemia (AML), and Stage IV Lymphoma. The following are not reported as Disseminated Cancer: Chronic Lymphocytic Leukemia (CLL), Chronic Myelogenous Leukemia (CML), Stages I through III Lymphomas or Multiple Myeloma. Example: A patient with a primary breast cancer with positive nodes in the axilla does NOT qualify for this definition. She has spread of the tumor to a site other than the primary site, but does not have widespread metastases. A patient with primary breast cancer with positive nodes in the axilla AND liver metastases does qualify, because she has both spread of the tumor to the axilla and other major organs.	Yes; No	NULL = Unknown
54	WNDINF	Char	Open wound/wound infection	Preoperative evidence of a documented open wound at the time of the principal operative procedure. An open wound is a breach in the integrity of the skin or separation of skin edges and includes open surgical wounds, with or without cellulitis or purulent exudate.<br>This does not include osteomyelitis or localized abscesses.<br>Assign Yes to:<br>- Open drains should be considered an open wound: (e.g. Penrose drains) - Open wounds currently undergoing dressing changes or with negative pressure wound devices (e.g., wound vacs) - Any abnormal passageway leading from an internal organ (e.g. intestinal tract) to the surface of the body / skin. (e.g. enterocutaneous fistula [ECF])<br>Assign No to:<br>- An ostomy would not be considered an open wound<br>- A scabbed over wound with or without drainage<br>- A Band-Aid over an open sore (break in skin)<br>- Oral sores<br>- A tracheostomy would not be considered an open wound	Yes; No	NULL = Unknown Definition revised or clarified from 2011
55	STEROID	Char	Steroid use for chronic condition	Patient has required the regular administration of oral or parenteral corticosteroid (e.g. Prednisone, Decadron) medications or immunosuppressant medications, within the 30 days prior to the principal operative procedure or at the time the patient is being considered as a candidate for surgery, for a chronic medical condition (e.g.<br>COPD, asthma, rheumatologic disease, rheumatoid arthritis, inflammatory bowel disease). A one-time pulse, limited short course, or a taper of less than 10 days duration would not qualify.<br>Do not include topical corticosteroids applied to the skin or corticosteroids administered by inhalation or rectally. Do not include patients who only receive short course steroids (duration 10 days or less) in the 30 days prior to surgery.	Yes; No	NULL = Unknown Definition revised or clarified from 2011
56	WTLOSS	Char	>10% loss body weight in last 6 months	"YES" is entered for patients with a greater than 10% decrease in body weight in the six month interval immediately preceding surgery as manifested by serial weights in the chart, as reported by the patient, or as evidenced by change in clothing size or severe cachexia. Patients who have intentionally lost weight as part of a weight reduction program do not qualify.	Yes; No	NULL = Unknown
57	BLEEDDIS	Char	Bleeding disorders	"YES" is entered for patients with any condition that places the patient at risk for excessive bleeding requiring hospitalization due to a deficiency of blood clotting elements (e.g., vitamin K deficiency, hemophilias, thrombocytopenia, chronic anticoagulation therapy that has not been discontinued prior to surgery). Patients not included who are on chronic aspirin therapy. If there is **no** documentation of **discontinuation** of medication, "YES" is entered for bleeding disorder.	Yes; No	NULL = Unknown
58	TRANSFUS	Char	Preop Transfusion of >= 1 unit of whole/packed RBCs in 72 hours prior to surgery	Preoperative loss of blood necessitating any transfusion (minimum of 1 unit) of whole blood/packed red cells transfused during the 72 hours prior to surgery start time, including any blood transfused in the emergency room. If greater than 200 units, enter 200 units.	Yes; No	NULL = Unknown

Position #	Variable Name	Data Type	Variable Label	Variable Definition	Variable Options at Entry	Comments
59	CHEMO	Char	Chemotherapy for malignancy in <= 30 days pre-op	"YES" entered if the patient had any chemotherapy treatment for cancer in the 30 days prior to surgery. Chemotherapy may include, but is not restricted to, oral and parenteral treatment with chemotherapeutic agents for malignancies such as colon, breast, lung, head and neck, and gastrointestinal solid tumors as well as lymphatic and hematopoietic malignancies such as lymphomas, leukemia, and multiple myeloma. Patient is not included if treatment consists solely of hormonal therapy. Chemotherapy treatment must be for malignancy.	Yes; No	NULL = Unknown Definition revised or clarified from 2010
60	RADIO	Char	Radiotherapy for malignancy in last 90 days	"YES" entered if the patient had any radiotherapy treatment for cancer in the 90 days prior to surgery. Count If the patient had radiation seeds implanted and the implantation was within 90 days prior to the operation.	Yes; No	NULL = Unknown
61	PRSEPIS	Char	Systemic Sepsis	Sepsis is a vast clinical entity that takes a variety of forms. The spectrum of disorders spans from relatively mild physiologic abnormalities to septic shock. The most significant level is reported using the following criteria: **SIRS** (Systemic Inflammatory Response Syndrome): SIRS is a widespread inflammatory response to a variety of severe clinical insults. This syndrome is clinically recognized by the presence of two or more of the following within the same time frame: Temp >38 degrees C or <36 degrees C HR >90 bpm RR >20 breaths/min or PaCO2 <32 mmHg(<4.3 kPa) WBC >12,000 cell/mm3, <4000 cells/mm3, or >10% immature (band) forms Anion gap acidosis: this is defined by either: [Na + K] – [CL + HCO3 (or serum CO2]. If this number is greater than 16, then an anion gap acidosis is present. Na – [CL + HCO3 (or serum CO2]. If this number is greater than 12, then An anion gap acidosis is present. *If anion gap lab values are performed at your facilities lab, ascertain which formula is utilized and follow guideline criteria.<br><br>**Sepsis**: Sepsis is the systemic response to infection. Report this variable if the patient has clinical signs and symptoms of SIRS listed above and meets either A or B: A. One of the following:<br>- Positive blood culture<br>- Clinical documentation of purulence or positive culture from any site for which there is documentation noting the site as the acute case of sepsis.<br>B. Suspected pre-operative clinical condition of infection, or bowel infarction, which leads to the surgical procedure. The findings during the Principal Operative Procedure must confirm this suspected diagnosis with one or more of the following: Confirmed infarcted bowel requiring resection, purulence in the operative site, enteric contents in the operative site, or positive intra-operative cultures. **Septic Shock**: Report this variable if the patient has sepsis AND documented organ and/or circulatory dysfunction. Examples of organ dysfunction include: oliguria, acute alteration in mental status, acute respiratory distress. Examples of circulatory dysfunction include: hypotension, requirement of inotropic or vasopressor agents.	SIRS; Sepsis; Septic Shock; None	NULL = Unknown Definition revised or clarified from 2011
62	Pregnancy	Char	Pregnancy	"YES" entered if pregnant. Pregnancy is determined by one of the following: . Administration of a blood or urine pregnancy test with a positive result . Visualization of the fetus by ultrasound . Indication of fetal heart rate by ultrasound or fetal heart monitoring Pregnancy takes approximately 40 weeks between the time of the last menstrual cycle and delivery.	Yes; No	NULL = Not applicable or not documented because variable was added in July 2006
63	PrOper30	Char	Prior Operation within 30 days	"YES" entered if the patient has had any major surgical procedure performed within 30 days prior to the assessed operation that would meet the following NSQIP criteria: Operation was performed utilizing general, spinal, or epidural anesthesia or operation performed included any of the following: carotid endarterectomy, inguinal hernia repair, parathyroidectomy, thyroidectomy, breast lumpectomy, or endovascular AAA repair Operation was not listed on the NSQIP CPT Exclusion list. Also **included** are any transplant procedures or trauma procedures if performed within 30 days prior to the assessed operation.	Yes; No	NULL = Not applicable or not documented because variable was added in July 2006
64	DPRNA	Num	Days from Na Preoperative Labs to Operation	Days from Na Preoperative Labs to Operation		-99 = Lab value not obtained or Unknown
65	DPRBUN	Num	Days from BUN Preoperative Labs to Operation	Days from BUN Preoperative Labs to Operation		-99 = Lab value not obtained or Unknown

Position #	Variable Name	Data Type	Variable Label	Variable Definition	Variable Options at Entry	Comments
66	DPRCREAT	Num	Days from Creatinine Preoperative Labs to Operation	Days from Creatinine Preoperative Labs to Operation		-99 = Lab value not obtained or Unknown
67	DPRALBUM	Num	Days from Albumin Preoperative Labs to Operation	Days from Albumin Preoperative Labs to Operation		-99 = Lab value not obtained or Unknown
68	DPRBILI	Num	Days from Bilirubin Preoperative Labs to Operation	Days from Bilirubin Preoperative Labs to Operation		-99 = Lab value not obtained or Unknown
69	DPRSGOT	Num	Days from SGOT Preoperative Labs to Operation	Days from SGOT Preoperative Labs to Operation		-99 = Lab value not obtained or Unknown
70	DPRALKPH	Num	Days from ALKPHOS Preoperative Labs to Operation	Days from ALKPHOS Preoperative Labs to Operation		-99 = Lab value not obtained or Unknown
71	DPRWBC	Num	Days from WBC Preoperative Labs to Operation	Days from WBC Preoperative Labs to Operation		-99 = Lab value not obtained or Unknown
72	DPRHCT	Num	Days from HCT Preoperative Labs to Operation	Days from HCT Preoperative Labs to Operation		-99 = Lab value not obtained or Unknown
73	DPRPLATE	Num	Days from PlateCount Preoperative Labs to Operation	Days from PlateCount Preoperative Labs to Operation		-99 = Lab value not obtained or Unknown
74	DPRPTT	Num	Days from PTT Preoperative Labs to Operation	Days from PTT Preoperative Labs to Operation		-99 = Lab value not obtained or Unknown
75	DPRPT	Num	Days from PT Preoperative Labs to Operation	Days from PT Preoperative Labs to Operation		-99 = Lab value not obtained or Unknown
76	DPRINR	Num	Days from INR Preoperative Labs to Operation	Days from INR Preoperative Labs to Operation		-99 = Lab value not obtained or Unknown
77	PRSODM	Num	Pre-operative serum sodium	Pre-operative serum sodium		-99 = Lab value not obtained or Unknown
78	PRBUN	Num	Pre-operative BUN	Pre-operative BUN		-99 = Lab value not obtained or Unknown
79	PRCREAT	Num	Pre-operative serum creatinine	Pre-operative serum creatinine		-99 = Lab value not obtained or Unknown
80	PRALBUM	Num	Pre-operative serum albumin	Pre-operative serum albumin		-99 = Lab value not obtained or Unknown
81	PRBILI	Num	Pre-operative total bilirubin	Pre-operative total bilirubin		-99 = Lab value not obtained or Unknown
82	PRSGOT	Num	Pre-operative SGOT	Pre-operative SGOT		-99 = Lab value not obtained or Unknown
83	PRALKPH	Num	Pre-operative alkaline phosphatase	Pre-operative alkaline phosphatase		-99 = Lab value not obtained or Unknown
84	PRWBC	Num	Pre-operative WBC	Pre-operative WBC		-99 = Lab value not obtained or Unknown
85	PRHCT	Num	Pre-operative hematocrit	Pre-operative hematocrit		-99 = Lab value not obtained or Unknown
86	PRPLATE	Num	Pre-operative platelet count	Pre-operative platelet count		-99 = Lab value not obtained or Unknown
87	PRPTT	Num	Pre-operative PTT	Pre-operative PTT		-99 = Lab value not obtained or Unknown
88	PRINR	Num	Pre-operative International Normalized Ratio (INR) of PT values	Pre-operative International Normalized Ratio (INR) of PT values		-99 = Lab value not obtained or Unknown
89	PRPT	Num	Pre-operative PT	Pre-operative PT		-99 = Lab value not obtained or Unknown
90	OTHERPROC1	Char	Other Procedure 1	An additional operative procedure performed by the **same surgical team** (i.e., the same specialty/service) **under the same anesthetic** which has a CPT code different from that of the Principal Operative Procedure (e.g., a splenectomy performed in the course of a cholecystectomy). **ALL additional procedures/CPT codes for the OR visit are reported.**		NULL = No Procedure
91	OTHERCPT1	Char	Other CPT Code 1	CPT Code		NULL = No Procedure
92	OTHERWRVU1	Num	Other Work Relative Value Unit 1	Other Work Relative Value Unit 1		-99 = No Procedure/Unknown
93	OTHERPROC2	Char	Other Procedure 2	See 'Other Procedure 1'		NULL = No Procedure
94	OTHERCPT2	Char	Other CPT Code 2	CPT Code		NULL = No Procedure
95	OTHERWRVU2	Num	Other Work Relative Value Unit 2	Other Work Relative Value Unit 2		-99 = No Procedure/Unknown
96	OTHERPROC3	Char	Other Procedure 3	See 'Other Procedure 1'		NULL = No Procedure
97	OTHERCPT3	Char	Other CPT Code 3	CPT Code		NULL = No Procedure
98	OTHERWRVU3	Num	Other Work Relative Value Unit 3	Other Work Relative Value Unit 3		-99 = No Procedure/Unknown
99	OTHERPROC4	Char	Other Procedure 4	See 'Other Procedure 1'		NULL = No Procedure
100	OTHERCPT4	Char	Other CPT Code 4	CPT Code		NULL = No Procedure
101	OTHERWRVU4	Num	Other Work Relative Value Unit 4	Other Work Relative Value Unit 4		-99 = No Procedure/Unknown
102	OTHERPROC5	Char	Other Procedure 5	See 'Other Procedure 1'		NULL = No Procedure
103	OTHERCPT5	Char	Other CPT Code 5	CPT Code		NULL = No Procedure
104	OTHERWRVU5	Num	Other Work Relative Value Unit 5	Other Work Relative Value Unit 5		-99 = No Procedure/Unknown

Position #	Variable Name	Data Type	Variable Label	Variable Definition	Variable Options at Entry	Comments
105	OTHERPROC6	Char	Other Procedure 6	See 'Other Procedure 1'		NULL = No Procedure
106	OTHERCPT6	Char	Other CPT Code 6	CPT Code		NULL = No Procedure
107	OTHERWRVU6	Num	Other Work Relative Value Unit 6	Other Work Relative Value Unit 6		-99 = No Procedure/Unknown
108	OTHERPROC7	Char	Other Procedure 7	See 'Other Procedure 1'		NULL = No Procedure
109	OTHERCPT7	Char	Other CPT Code 7	CPT Code		NULL = No Procedure
110	OTHERWRVU7	Num	Other Work Relative Value Unit 7	Other Work Relative Value Unit 7		-99 = No Procedure/Unknown
111	OTHERPROC8	Char	Other Procedure 8	See 'Other Procedure 1'		NULL = No Procedure
112	OTHERCPT8	Char	Other CPT Code 8	CPT Code		NULL = No Procedure
113	OTHERWRVU8	Num	Other Work Relative Value Unit 8	Other Work Relative Value Unit 8		-99 = No Procedure/Unknown
114	OTHERPROC9	Char	Other Procedure 9	See 'Other Procedure 1'		NULL = No Procedure
115	OTHERCPT9	Char	Other CPT Code 9	CPT Code		NULL = No Procedure
116	OTHERWRVU9	Num	Other Work Relative Value Unit 9	Other Work Relative Value Unit 9		-99 = No Procedure/Unknown
117	OTHERPROC10	Char	Other Procedure 10	See 'Other Procedure 1'		NULL = No Procedure
118	OTHERCPT10	Char	Other CPT Code 10	CPT Code		NULL = No Procedure
119	OTHERWRVU10	Num	Other Work Relative Value Unit 10	Other Work Relative Value Unit 10		-99 = No Procedure/Unknown
120	CONCURR1	Char	Concurrent Procedure 1	An additional operative procedure performed by a different surgical team or surgeon (e.g., under direction of a different surgical attending) and under the same anesthetic which have CPT codes different* from that of the Principal Operative Procedure (for example, Coronary Artery Bypass Graft procedure on a patient who is also undergoing a Carotid Endarterectomy).<br>*Certain CPT codes can be billed for a patient more than one time reflecting repeated performance of a particular procedure. In such cases the codes could be considered different.		NULL = No Procedure<br>Definition revised or clarified from 2011
121	CONCPT1	Char	Concurrent CPT 1	Concurrent CPT 2		NULL = No Procedure
122	CONWRVU1	Num	Concurrent Work Relative Value Unit 1	Concurrent Work Relative Value Unit 2		-99 = No Procedure/Unknown
123	CONCURR2	Char	Concurrent Procedure 2	Concurrent Procedure 3		NULL = No Procedure
124	CONCPT2	Char	Concurrent CPT 2	Concurrent CPT 3		NULL = No Procedure
125	CONWRVU2	Num	Concurrent Work Relative Value Unit 2	Concurrent Work Relative Value Unit 3		-99 = No Procedure/Unknown
126	CONCURR3	Char	Concurrent Procedure 3	Concurrent Procedure 4		NULL = No Procedure
127	CONCPT3	Char	Concurrent CPT 3	Concurrent CPT 4		NULL = No Procedure
128	CONWRVU3	Num	Concurrent Work Relative Value Unit 3	Concurrent Work Relative Value Unit 4		-99 = No Procedure/Unknown
129	CONCURR4	Char	Concurrent Procedure 4	Concurrent Procedure 5		NULL = No Procedure
130	CONCPT4	Char	Concurrent CPT 4	Concurrent CPT 5		NULL = No Procedure
131	CONWRVU4	Num	Concurrent Work Relative Value Unit 4	Concurrent Work Relative Value Unit 5		-99 = No Procedure/Unknown
132	CONCURR5	Char	Concurrent Procedure 5	Concurrent Procedure 6		NULL = No Procedure
133	CONCPT5	Char	Concurrent CPT 5	Concurrent CPT 6		NULL = No Procedure
134	CONWRVU5	Num	Concurrent Work Relative Value Unit 5	Concurrent Work Relative Value Unit 6		-99 = No Procedure/Unknown
135	CONCURR6	Char	Concurrent Procedure 6	Concurrent Procedure 7		NULL = No Procedure
136	CONCPT6	Char	Concurrent CPT 6	Concurrent CPT 7		NULL = No Procedure
137	CONWRVU6	Num	Concurrent Work Relative Value Unit 6	Concurrent Work Relative Value Unit 7		-99 = No Procedure/Unknown
138	CONCURR7	Char	Concurrent Procedure 7	Concurrent Procedure 8		NULL = No Procedure
139	CONCPT7	Char	Concurrent CPT 7	Concurrent CPT 8		NULL = No Procedure
140	CONWRVU7	Num	Concurrent Work Relative Value Unit 7	Concurrent Work Relative Value Unit 8		-99 = No Procedure/Unknown
141	CONCURR8	Char	Concurrent Procedure 8	Concurrent Procedure 9		NULL = No Procedure
142	CONCPT8	Char	Concurrent CPT 8	Concurrent CPT 9		NULL = No Procedure
143	CONWRVU8	Num	Concurrent Work Relative Value Unit 8	Concurrent Work Relative Value Unit 9		-99 = No Procedure/Unknown
144	CONCURR9	Char	Concurrent Procedure 9	Concurrent Procedure 10		NULL = No Procedure
145	CONCPT9	Char	Concurrent CPT 9	Concurrent CPT 10		NULL = No Procedure
146	CONWRVU9	Num	Concurrent Work Relative Value Unit 9	Concurrent Work Relative Value Unit 10		-99 = No Procedure/Unknown
147	CONCURR10	Char	Concurrent Procedure 10	Concurrent Procedure 11		NULL = No Procedure
148	CONCPT10	Char	Concurrent CPT 10	Concurrent CPT 11		NULL = No Procedure
149	CONWRVU10	Num	Concurrent Work Relative Value Unit 10	Concurrent Work Relative Value Unit 11		-99 = No Procedure/Unknown
150	OPNOTE	Char	Surgeon who dictated the operative note.	Surgeon who dictated the operative note.	Attending<br>Resident<br>Not Available	Historical variable, no longer used
151	PGY	Num	Highest Level of Resident Surgeon	Report the highest Post-Graduate Year (PGY) of the resident(s) who scrubbed for the surgical procedure. Choose from 1 – 10. Enter '0' if there is no resident scrubbed on the surgical procedure.	0-10	-99 = Unknown

Position #	Variable Name	Data Type	Variable Label	Variable Definition	Variable Options at Entry	Comments
152	EMERGNCY	Char	Emergency case	Emergency Case: An emergency case is usually performed within a short interval of time between patient diagnosis or the onset of related preoperative symptomatology. It is implied that the patient's well-being and outcome is potentially threatened by unnecessary delay and the patient's status could deteriorate unpredictably or rapidly. The NSQIP Principal Operative Procedure must be performed during the hospital admission for the diagnosis. Patients who are discharged after diagnosis and return for an elective, semi-elective, or urgent procedure related to the diagnosis would not be considered to have had an emergent case. The intent is to identify a patient population with heightened surgical risk due to an ongoing acute process that is currently having a negative impact on the patients' health and for which continued, potentially rapid deterioration could occur. The increased risk might be partly due to the fact that the procedure is being performed with limited preoperative preparation time and the surgical team does not necessarily have the ability to optimize the patient's status. The emergency case variable distinguishes between urgent/semi-elective/elective cases and true emergent surgeries. Urgent/semi-elective cases are not considered emergencies. Assign 'YES' if the surgeon and/or anesthesiologist report the case as emergent.	Yes; No	NULL = Unknown Definition revised or clarified from 2011
153	WNDCLAS	Char	Wound classification	Wound classification should be assigned based on the primary principal procedure being performed. Wound class is not assigned based on an 'other' or 'concurrent' procedure. This variable indicates whether the primary surgeon has classified the wound as: **(1) Clean:** An uninfected operative wound in which no inflammation is encountered and the respiratory, alimentary, genital, or uninfected urinary tract is not entered. In addition, clean wounds are primarily closed and, if necessary, drained with closed drainage. Operative incisional wounds that follow nonpenetrating (blunt) trauma should be included in this category if they meet the criteria. *Examples of "Clean" cases include mastectomy, vascular bypass graft, exploratory laparotomy, hernia repair, thyroidectomy, total hip or knee replacement, total hip replacements for avascular necrosis, removal of 'old' hardware without evidence of infection. Note: Placement of any drain at the time of surgery does not change the classification of the wound.* **(2) Clean/Contaminated:** An operative wound in which the respiratory, alimentary, genital, or urinary tracts are entered under controlled conditions and without unusual contamination. Specifically, operations involving the biliary tract, appendix, vagina, and oropharynx are included in this category, provided no evidence of infection or major break in technique is encountered. *Examples of "Clean/Contaminated" cases include cholecystectomy, colectomy, colostomy reversals, roux-en-Y, laryngectomy, small bowel resection, transurethral resection of the prostate, Whipple pancreaticoduodenectomy.* **(3) Contaminated:** Open, fresh, accidental wounds. In addition, operations with major breaks in sterile technique or gross spillage from the gastrointestinal tract, and incisions in which acute, nonpurulent inflammation is encountered including necrotic tissue without evidence of purulent drainage (e.g. dry gangrene) are included in this category. *Examples of "Contaminated" cases include appendectomy for inflamed appendicitis, bile spillage during cholecystectomy, or open cardiac massage. Open surgical wounds returning to the OR. Examples of major break in sterile technique include but are not limited to non-sterile equipment or debris found in the operative field.* **(4) Dirty/Infected:** Old traumatic wounds with retained devitalized tissue and those that involve existing clinical infection or perforated viscera. This definition suggests that the organisms causing postoperative infection were present in the operative field before the operation.	1-Clean<br>2-Clean/Contaminated<br>3-Contaminated<br>4-Dirty/Infected	NULL = Unknown Definition revised or clarified from 2011

Position #	Variable Name	Data Type	Variable Label	Variable Definition	Variable Options at Entry	Comments
	WNDCLAS (Continued)	Char	Wound classification	Examples of "Dirty/Infected" cases include excision and drainage of abscess, perforated bowel, peritonitis, ruptured appendix.<br>Wound Class for Non-Skin Incision Surgeries (Natural Orifice): assign the wound classification based on which orifice was entered. Example: appendectomy performed via the vagina would, at minimum, be a clean/contaminated wound class. Multiple surgical procedures performed with different incision sites = Assign wound classification based on the Principal Operative Procedure being reviewed in NSQIP.<br>Revision: January 1, 2012 ACS 4-24 NSQIP<br>Example:<br>Principal Operative Procedure: Carotid Endarterectomy (clean) Other Procedure: I & D of an infected right big toe (dirty/infected). The wound class assigned to this case would be clean.<br>Multiple surgical procedures performed through one incision (same operative space) = Assign wound classification based on the assessment of the overall operative space.<br>Example:<br>Principal Operative Procedure: Lysis of adhesions (clean) Other Procedure: cholecystectomy with gross bile spillage (contaminated). The wound class would be contaminated, as the spillage is in the same operative space as the Principal Operative Procedure.		
154	ASACLAS	Char	ASA classification	The American Society of Anesthesiology (ASA) Physical Status Classification of the patient's present physical condition on a scale from 1-5 as it appears on the anesthesia record. The classifications are as follows: **ASA 1** -Normal healthy patient **ASA 2** -Patient with mild systemic disease **ASA 3** -Patient with severe systemic disease **ASA 4** -Patient with severe systemic disease that is a constant threat to life **ASA 5** -Moribund patient who is not expected to survive without the operation.	1 -No Disturb<br>2 -Mild Disturb<br>3 -Severe Disturb<br>4 -Life Threat<br>5 -Moribund<br>None assigned	NULL= Unknown
155	AIRTRA	Char	Airway trauma	The code corresponding to trauma resulting from the endotracheal intubation process is entered.	None<br>Lip laceration or hematoma<br>Tooth chipped, loosened or lost<br>Tongue laceration or hematoma<br>Pharyngeal laceration<br>Laryngeal laceration<br>Failure to intubate	Historical variable, no longer used
156	MALLAMP	Num	Mallampati scale	The Mallampati classification relates tongue size to pharyngeal size. This test is performed with the patient in sitting position, the head held in a neutral position, the mouth wide open, and the tongue protruding to the maximum. The subsequent classification is assigned based upon the pharyngeal structures that are visible: **Class I** – visualization of the soft palate, fauces, uvula, and anterior and posterior pillars. **Class II** – visualization of the soft palate, fauces, and uvula. **Class III** – visualization of the soft palate and the base of the uvula. **Class IV** – soft palate is not visible at all.	1; 2; 3; 4	Historical variable, no longer used
157	MORTPROB	Num	Estimated Probability of Mortality	Probability of mortality is developed for general and vascular surgical cases based on a logistic regresion analysis using the patient's preopeartive characteristics as the independent or predictive variables. Only general and vascular cases used in the logistic regression analysis will have the associated probabilities of mortality.		System missing = case was not included in the logistic regression analysis
158	MORBPROB	Num	Estimated Probability of Morbidity	Probability of morbidity is developed for general and vascular surgical cases based on a logistic regresion analysis using the patient's preopeartive characteristics as the independent or predictive variables. Only the general and vascular cases used in the logistic regression analysis will have the associated probabilities of morbidity.		System missing = case was not included in the logistic regression analysis
159	RBC	Num	Number of RBC units given intraoperative	The number of packed or whole red blood cells given during the operative procedure as it appears on the anesthesia record. The amount of blood reinfused from the cell saver is also noted. For a cell saver, every 500 cc's of fluid will equal 1 unit of packed cells. If there is less than 250 cc of fluid, 0 is entered.		Historical variable, no longer used
160	ANESURG	Num	Duration from Anesthsia start to Surgery start	Duration from Anesthsia start to Surgery start in minutes		-99 = Unknown
161	SURGANE	Num	Duration from Surgery stop to Anesthia Stop	Duration from Surgery stop to Anesthia Stop in minutes		-99 = Unknown

Position #	Variable Name	Data Type	Variable Label	Variable Definition	Variable Options at Entry	Comments
162	DPATRM	Num	Duration patient is in Room	Duration patient is in Room in minutes		-99 = Unknown
163	ANETIME	Num	Duration of Anesthesia	Duration of Anesthesia in minutes		-99 = Unknown
164	OPTIME	Num	Total operation time	Total operation time in minutes		-99 = Unknown
165	TYPEINTOC	Char	Type of Intraoperative Occurrence	One of the three following intraoperative occurrences can be selected. Cardiac Arrest Requiring CPR is defined as the absence of cardiac rhythm or presence of chaotic cardiac rhythm that results in loss of consciousness requiring the initiation of any component of basic and/or advanced cardiac life support. Patients with automatic implantable cardioverter defibrillator that fire but the patient has no loss of consciousness should be excluded. Myocardial Infarction is defined as a new transmural acute myocardial infarction occurring during surgery as manifested by nnew Q-waves on ECG. Unplanned Intubation for Respirator/Cardiac Failure is defined as a patient requiring placement of an endotracheal tube or other similar breathing tube [Laryngeal Mask Airway (LMA), nasotracheal tube, etc] and ventilator support which was not intended or planned.	Cardiac Arrest Requiring CPR<br>Myocardial Infarction<br>Unplanned Intubation	NULL = None of the three occurred
166	SDISDT	Num	Year discharged/transferred from surgical service	Year discharged/transferred from surgical service		Historical variable, no longer used
167	HDISDT	Num	Hospital discharge Year	Hospital discharge Year		
168	YRDEATH	Num	Year of death	Year of death		-99 = Patient alive at 30 days
169	TOTHLOS	Num	Length of total hospital stay	Length of total hospital stay		
170	AdmQtr	Num	Quarter of Admission	Quarter of Admission	1; 2; 3; 4	-99 = Unknown
171	HtoODay	Num	Days from Hospital Admission to Operation	Days from Hospital Admission to Operation		-99 = Unknown
172	StoODay	Num	Days from Surgical Admission to Operation	Days from Surgical Admission to Operation		Historical variable, no longer used
173	TOTSLOS	Num	Length of total surgical stay	Length of total surgical stay		Historical variable, no longer used
174	NSUPINFEC	Num	Number of Wound Occurrences	Number of Superficial Wound Occurrences		
175	SUPINFEC	Char	Superficial surgical site infection	Superficial incisional SSI is an infection that occurs within 30 days after the operation and the infection involves only skin or subcutaneous tissue of the incision and at least one of the following: -Purulent drainage, with or without laboratory confirmation, from the superficial incision. -Organisms isolated from an aseptically obtained culture of fluid or tissue from the superficial incision. -At least one of the following signs or symptoms of infection: pain or tenderness, localized swelling, redness, or heat AND superficial incision is deliberately opened by the surgeon, unless incision is culture-negative. -Diagnosis of superficial incisional SSI by the surgeon or attending physician. Do not report the following conditions as SSI: -Stitch abscess (minimal inflammation and discharge confined to the points of suture penetration). -Infected burn wound. -Incisional SSI that extends into the fascial and muscle layers (see deep incisional SSI).	No Complication; Superficial Incisional SSI	
176	SSSIPATOS	Char	Superficial Incisional SSI PATOS	If a 'Superficial Incisional SSI' is noted as a postoperative outcome, and an open wound, cellulitis (erythema, tenderness AND swelling), or wound infection was noted preoperatively or intraoperatively at the surgical site at the time of surgery; select 'YES'. Guidance: if a Superficial Incisional SSI is assigned as a postoperative occurrence -- only Superficial Incisional SSI PATOS can be assigned if the patient meets criteria for Superficial Incisional PATOS [Cannot assign Deep or Organ/Space PATOS]	Yes; No	NULL = No response Variable added in 2011
177	DSUPINFEC	Num	Days from Operation until Superficial Incisional SSI Complication	Days from Operation until Superficial Incisional SSI Complication		-99 = Patient did not experience this complication at or before 30 days post operation
178	NWNDINFD	Num	Number of Deep Incisional SSI Occurrences	Number of Deep Incisional SSI Occurrences		

Position #	Variable Name	Data Type	Variable Label	Variable Definition	Variable Options at Entry	Comments
179	WNDINFD	Char	Occurrences Deep Incisional SSI	Deep Incision SSI is an infection that occurs within 30 days after the operation and the infection appears to be related to the operation and infection involved deep soft tissues (e.g., fascial and muscle layers) of the incision and at least one of the following: -Purulent drainage from the deep incision but not from the organ/space component of the surgical site. -A deep incision spontaneously dehisces or is deliberately opened by a surgeon when the patient has at least one of the following signs or symptoms: fever (> 38 C), localized pain, or tenderness, unless site is culture-negative. -An abscess or other evidence of infection involving the deep incision is found on direct examination, during reoperation, or by histopathologic or radiologic examination. -Diagnosis of a deep incision SSI by a surgeon or attending physician. Note: -Infection that involves both superficial and deep incision sites is reported as deep incisional SSI. -An organ/space SSI that drains through the incision is reported as a deep incisional SSI.	Deep Incisional SSI; No Complication	
180	DSSIPATOS	Char	Deep Incisional SSI PATOS	If a 'Deep Incisional SSI' is noted as a postoperative outcome, and an open wound, cellulitis (erythema, tenderness AND swelling) or infection was noted preoperatively or intaoperatively at the surgical site at the time of surgery; select 'YES'. Guidance: if a Deep Incisional SSI is assigned as a postoperative occurrence -- only Deep Incisional SSI PATOS can be assigned if the patient meets criteria for Deep Incisional SSI PATOS [Cannot assign Superficial or Organ/Space PATOS]	Yes; No	NULL = No response Variable added in 2011
181	DWNDINFD	Num	Days from Operation until Deep Incisional SSI Complication	Days from Operation until Deep Incisional SSI Complication		-99 = Patient did not experience this complication at or before 30 days post operation
182	NORGSPCSSI	Num	Number of Organ/Space SSI Occurrences	Number of Organ/Space SSI Occurrences		
183	ORGSPCSSI	Char	Occurrences Organ Space SSI	Organ/Space SSI is an infection that occurs within 30 days after the operation and the infection appears to be related to the operation and the infection involves any part of the anatomy (e.g., organs or spaces), other than the incision, which was opened or manipulated during an operation and at least one of the following: Purulent drainage from a drain that is placed through a stab wound into the organ/space. -Organisms isolated from an aseptically obtained culture of fluid or tissue in the organ/space. -An abscess or other evidence of infection involving the organ/space that is found on direct examination, during reoperation, or by histopathologic or radiologic examination. -Diagnosis of an organ/space SSI by a surgeon or attending physician.	Organ/Space SSI; No Complication	
184	OSSIPATOS	Char	Organ/Space SSI PATOS	If an 'Organ / Space SSI' is noted as a postoperative outcome, and an abscess or other evidence of infection involving the organ/space was noted preoperatively or intraoperatively at the surgical area at the time of surgery; select 'YES' for this variable. Guidance: if an Organ/Space SSI is assigned as a postoperative occurrence—only Organ/Space SSI PATOS can be assigned if the patient meets criteria for Organ/Space SSI PATOS [Cannot assign Superficial or Deep PATOS]	Yes; No	NULL = No response Variable added in 2011
185	DORGSPCSSI	Num	Days from Operation until Organ/Space SSI Complication	Days from Operation until Organ/Space SSI Complication		-99 = Patient did not experience this complication at or before 30 days post operation
186	NDEHIS	Num	Number of Wound Disruption Occurrences	Number of Wound Disruption Occurrences		
187	DEHIS	Char	Occurrences Wound Disrupt	Abdominal site: refers primarily to loss of the integrity of fascial closure (or whatever closure was performed in the absence of fascial closure). Other Surgical Sites: there must be a total breakdown of the surgical closure compromising the integrity of the procedure. Example: tissue flap coverage where the surgical incisions, which were closed, have lost the integrity of closure. An ostomy with a small separation around it would NOT qualify..	Wound Disruption; No complication	Definition revised or clarified from 2011

Position #	Variable Name	Data Type	Variable Label	Variable Definition	Variable Options at Entry	Comments
188	DDEHIS	Num	Days from Operation until Wound Disruption Complication	Days from Operation until Wound Disruption Complication		-99 = Patient did not experience this complication at or before 30 days post operation
189	NOUPNEUMO	Num	Number of Pneumonia Occurrences	Number of Pneumonia Occurrences		
190	OUPNEUMO	Char	Occurrences Pneumonia	Enter "Yes" if the patient has pneumonia meeting the definition below. Patients with pneumonia *must meet criteria from both* Radiology *and* Signs/Symptoms/Laboratory sections listed as follows:<br><br>Radiology:<br>One definitive chest radiological exam (x-ray or CT)* with at least one of the following:<br>•New or progressive and persistent infiltrate<br>•Consolidation or opacity<br>•Cavitation<br>Note: In patients with underlying pulmonary or cardiac disease (e.g. respiratory distress syndrome, bronchopulmonary dysplasia, pulmonary edema, or chronic obstructive pulmonary disease), two or more serial chest radiological exams (x-ray or CT) are required. (Serial radiological exams should be taken no less than 12 hours apart, but not more than 7 days apart. The occurrence should be assigned on the date the patient first met all of the criteria of the definition (i.e, if the patient meets all PNA criteria on the day of the first xray, assign this date to the occurrence. Do not assign the date of the occurrence to when the second serial xray was performed).<br><br>Signs/Symptoms/Laboratory:<br>FOR ANY PATIENT, at least one of the following:<br>•Fever (>38 C or >100.4 F) with no other recognized cause<br>•Leukopenia (<4000 WBC/mm3) or leukocytosis(≥12,000 WBC/mm3)<br>•For adults ≥ 70 years old, altered mental status with no other recognized cause<br>And<br>At least one of the following:<br>•5% Bronchoalveolar lavage (BAL) -obtained cells contain intracellular bacteria on direct microscopic exam (e.g., Gram stain)<br>•Positive growth in blood culture not related to another source of infection<br>•Positive growth in culture of pleural fluid<br>•Positive quantitative culture from minimally contaminated lower respiratory tract (LRT) specimen (e.g. BAL or protected specimen brushing)<br>OR<br>At least two of the following:<br>•New onset of purulent sputum, or change in character of sputum, or increased respiratory secretions, or increased suctioning requirements<br>•New onset or worsening cough, or dyspnea, or tachypnea<br>•Rales or rhonchi<br>•Worsening gas exchange (e.g. O2 desaturations (e.g., PaO2/FiO2 ≤ 240), increased oxygen requirements, or increased ventilator demand)	Pneumonia; No complication	Definition revised or clarified from 2010
191	PNAPATOS	Char	Pneumonia PATOS	If pneumonia is noted as a postoperative outcome, and documented as a preoperative risk factor; select 'YES' for this variable. Also, select 'YES (for PATOS only) if preoperative data are highly suggestive or suspicious of pneumonia.	Yes; No	NULL = No response Variable added in 2011
192	DOUPNEUMO	Num	Days from Operation until Pneumonia Complication	Days from Operation until Pneumonia Complication		-99 = Patient did not experience this complication at or before 30 days post operation (One case with a pneumonia complication had an unknown date within 30 days and thus the duration was set to -99)
193	NREINTUB	Num	Number of Unplanned Intubation Occurrences	Number of Unplanned Intubation Occurrences		

Position #	Variable Name	Data Type	Variable Label	Variable Definition	Variable Options at Entry	Comments
194	REINTUB	Char	Occurrences Unplanned Intubation	Patient required placement of an endotracheal tube or other similar breathing tube [Laryngeal Mask Airway (LMA), nasotracheal tube, etc] and ventilator support intraoperatively or within 30 days following surgery which was not intended or planned.<br>•The variable intent is to capture all cause unplanned intubations, including but not limited to unplanned intubations for refractory hypotension, cardiac arrest, inability to protect airway.<br>•Accidental self extubations requiring reintubation would be assigned.<br>•Emergency tracheostomy would be assigned. Patients with a chronic/long-term tracheostomy who are on and off the ventilator would not be assigned, unless the tracheostomy tube itself is removed and the patient requires reintubation (endotracheal or a new tracheostomy tube) or an emergency tracheostomy.<br>•Patients undergoing time off the ventilator during weaning trials and who fail the trail and are placed back on the ventilator would not be assigned.<br>•Intubations for an unplanned return to the OR would not be assigned, as the intubation is planned, it is the return to the OR which is unplanned.<br>•In patients who were intubated for a return to the OR for a surgical procedure unplanned intubation occurs after they have been extubated after surgery. In patients who were not intubated for a return to the OR, intubation at any time after their surgery is complete is considered unplanned.<br>•Intraoperative conversion from local or MAC anesthesia to general anesthesia, during the Principal Operative Procedure, with placement of a breathing tube and ventilator support, secondary to the patient not tolerating local or MAC anesthesia, in the absence of an emergency, would not be assigned.	Unplanned Intubation; No Complication	Definition revised or clarified from 2011
195	DREINTUB	Num	Days from Operation until Unplanned Intubation Complication	Days from Operation until Unplanned Intubation Complication		-99 = Patient did not experience this complication at or before 30 days post operation
196	NPULEMBOL	Num	Number of Pulmonary Embolism Occurrences	Number of Pulmonary Embolism Occurrences		
197	PULEMBOL	Char	Occurrences Pulmonary Embolism	Lodging of a blood clot in the pulmonary artery with subsequent obstruction of blood supply to the lung parenchyma. The blood clots usually originate from the deep leg veins or the pelvic venous system.<br>The identification of a new blood clot in a pulmonary artery causing obstruction (complete or partial) of the blood supply to the lungs. However, since there are not always preoperative studies proving that a clot or thrombus was not present preoperatively, the technical specification of the variable requires only a "new diagnosis"- in other words the clot or thrombus was not previously known.<br>A pulmonary embolism must be noted within 30 days after the principal operative procedure AND the following criteria, A AND B below:<br>A. New diagnosis of a new blood clot in a pulmonary artery<br>AND B. The patient has a V-Q scan interpreted as high probability of pulmonary embolism or a positive CT exam, TEE, pulmonary arteriogram, CT angiogram, or any other definitive imaging modality (including direct pathology examination such as autopsy	Pulmonary Embolism; No Complication	Definition revised or clarified in 2013
198	DPULEMBOL	Num	Days from Operation until Pulmonary Embolism Complication	Days from Operation until Pulmonary Embolism Complication		-99 = Patient did not experience this complication at or before 30 days post operation
199	NFAILWEAN	Num	Number of On Ventilator > 48 Hours Occurrences	Number of On Ventilator > 48 Hours Occurrences		
200	FAILWEAN	Char	Occurrences Ventilator > 48Hours	Total duration of ventilator-assisted respirations during postoperative hospitalization was greater than 48 hours. This can occur at any time during the 30-day period postoperatively. This time assessment is CUMULATIVE, not necessarily consecutive. Ventilator-assisted respirations can be via endotracheal tube, nasotracheal tube, or tracheostomy tube.	On Ventilator greater than 48 Hours; No Complication	
201	VENTPATOS	Char	On Ventilator > 48 Hours PATOS	If 'On Ventilator > 48 hours' is selected as a postoperative outcome, and patient is intubated prior to arrival to the OR; select 'YES'.	Yes; No	NULL = No response Variable added in 2011

Position #	Variable Name	Data Type	Variable Label	Variable Definition	Variable Options at Entry	Comments
202	DFAILWEAN	Num	Days from Operation until On Ventilator > 48 Hours Complication	Days from Operation until On Ventilator > 48 Hours Complication		-99 = Patient did not experience this complication at or before 30 days post operation (One case with a fail to wean complication had an unknown date within 30 days and thus the duration was set to -99)
203	NRENAINSF	Num	Number of Progressive Renal Insufficiency Occurrences	Number of Progressive Renal Insufficiency Occurrences		
204	RENAINSF	Char	Occurrences Progressive Renal Insufficiency	The reduced capacity of the kidney to perform its function as evidenced by a rise in creatinine of >2 mg/dl from preoperative value, but with no requirement for dialysis within 30 days of the operation.	Progressive Renal Insufficiency; No Complication	
205	DRENAINSF	Num	Days from Operation until Progressive Renal Insufficiency Complication	Days from Operation until Progressive Renal Insufficiency Complication		-99 = Patient did not experience this complication at or before 30 days post operation
206	NOPRENAFL	Num	Number of Acute Renal Failure Occurrences	Number of Acute Renal Failure Occurrences		
207	OPRENAFL	Char	Occurrences Acute Renal Fail	A patient who did not require dialysis preoperatively, worsening of renal dysfunction postoperatively requiring hemodialysis, peritoneal dialysis, hemofiltration, hemodiafiltration, or ultrafiltration.<br>- If the patient refuses a recommendation for dialysis, you would answer 'Yes' to this variable because the patient required dialysis -<br>Hemodialysis, peritoneal dialysis, hemofiltration, hemodiafiltration, or ultrafiltration all qualify -<br>Placement of a dialysis catheter is indicative of the need for dialysis, if used within 48 hours of placement	Acute Renal Failure; No Complication	Definition revised or clarified from 2011
208	DOPRENAFL	Num	Days from Operation until Acute Renal Failure Complication	Days from Operation until Acute Renal Failure Complication		-99 = Patient did not experience this complication at or before 30 days post operation
209	NURNINFEC	Num	Number of Urinary Tract infection Occurrences	Number of Urinary Tract infection Occurrences		
210	URNINFEC	Char	Occurrences Urinary Tract Infection	An infection in the urinary tract (kidneys, ureters, bladder, and urethra)<br>Must be noted within 30 days after the principal operative procedure AND patient must meet ONE of the following A OR B below:<br>A: ONE of the following six criteria: fever (>38oC or 100.4o F) ;urgency ;frequency ;dysuria ;suprapubic ;tenderness ;costovertebral angle pain or tenderness<br>AND A urine culture of > 100,000 colonies/ml urine with no more than two species of organisms<br>OR B: TWO of the following six criteria: fever (>38o C or 100.4o F) ;urgency ;frequency ;dysuria ;suprapubic tenderness ;costovertebral angle pain or tenderness<br>AND<br>At least one of the following: Dipstick test positive for leukocyte esterase and/or nitrate ;Pyuria (>10 WBCs/mm3 or > 3 WBC/hpf of unspun urine) ;Organisms seen on Gram stain of unspun urine ;Two urine cultures with repeated isolation of the same uropathogen with >100,000 colonies/ml urine in non-voided specimen ;Urine culture with < 100,000 colonies/ml urine of single uropathogen in patient being treated with appropriate antimicrobial therapy ;Physician's diagnosis ;Physician institutes appropriate antimicrobial therapy	Urinary Tract Infection; No Complication	Definition revised or clarified from 2013
211	UTIPATOS	Char	UTI PATOS	If a UTI is noted as a postoperative outcome, and there was any preoperative evidence of a symptomatic UTI (that had not started treatment or is currently undergoing treatment) or preoperative evidence was highly suggestive or suspicious of a UTI at the time of surgery; select 'YES'.	Yes; No	NULL = No Response Variable added in 2011
212	DURNINFEC	Num	Days from Operation until Urinary Tract Infection Complication	Days from Operation until Urinary Tract Infection Complication		-99 = Patient did not experience this complication at or before 30 days post operation (One case with a UTI complication had an unknown date within 30 days and thus the duration was set to -99)
213	NCNSCVA	Num	Number of Stroke/CVA Occurrences	Number of Stroke/CVA Occurrences		

Position #	Variable Name	Data Type	Variable Label	Variable Definition	Variable Options at Entry	Comments
214	CNSCVA	Char	CVA/Stroke with neurological deficit	Patient develops an embolic, thrombotic, or hemorrhagic vascular accident or stroke with motor, sensory, or cognitive dysfunction (e.g., hemiplegia, hemiparesis, aphasia, sensory deficit, impaired memory) that persists for 24 or more hours. If a specific time frame for the dysfunction is not documented in the medical record, but there is a diagnosis of a stroke, assign the occurrence, unless documentation specifically states that the motor, sensory, or cognitive dysfunction resolved.	Stroke/CVA; No Complication	Definition revised or clarified from 2010
215	DCNSCVA	Num	Days from Operation until Stroke/CVA Complication	Days from Operation until Stroke/CVA Complication		-99 = Patient did not experience this complication at or before 30 days post operation
216	NCNSCOMA	Num	Number of Coma > 24 Hours Occurrences	Number of Coma > 24 Hours Occurrences		
217	CNSCOMA	Char	Coma >24 hours	Patient is unconscious, or postures to painful stimuli, or is unresponsive to all stimuli (exclude transient disorientation or psychosis) for greater than 24 hours. Drug-induced coma (e.g. Propofol drips) are not entered within 30 days of the operation.	Coma greater than 24 hours; No Complication	
218	DCNSCOMA	Num	Days from Operation until Coma > 24 Hours Complication	Days from Operation until Coma > 24 Hours Complication		-99 = Patient did not experience this complication at or before 30 days post operation
219	NNEURODEF	Num	Number of Peripheral Nerve Injury Occurrences	Number of Peripheral Nerve Injury Occurrences		
220	NEURODEF	Char	Peripheral Nerve Injury	Peripheral nerve damage may result from damage to the nerve fibers, cell body, or myelin sheath during surgery. Peripheral nerve injuries which result in motor deficits to the cervical plexus, brachial plexus, ulnar plexus, lumbar-sacral plexus (sciatic nerve), peroneal nerve, and/or the femoral nerve should be included.	Peripheral nerve injury ; No Complication	
221	DNEURODEF	Num	Days from Operation until Peripheral Nerve Injury Complication	Days from Operation until Peripheral Nerve Injury Complication		-99 = Patient did not experience this complication at or before 30 days post operation
222	NCDARREST	Num	Number of Cardiac Arrest Requiring CPR Occurrences	Number of Cardiac Arrest Requiring CPR Occurrences		
223	CDARREST	Char	Occurrences Cardiac Arrest Requiring CPR	The absence of cardiac rhythm or presence of chaotic cardiac rhythm, intraoperatively or within 30 days following surgery, which results in a cardiac arrest requiring the initiation of CPR, which includes chest compressions. Patients are included who are in a pulseless VT or Vfib in which defibrillation is performed and PEA arrests requiring chest compressions. Patients with automatic implantable cardioverter defibrillator (AICD) that fire but the patient has no loss of consciousness should be excluded.	Cardiac Arrest Requiring CPR; No Complication	Definition revised or clarified from 2011
224	DCDARREST	Num	Days from Operation until Cardiac Arrest Requiring CPR Complication	Days from Operation until Cardiac Arrest Requiring CPR Complication		-99 = Patient did not experience this complication at or before 30 days post operation
225	NCDMI	Num	Number of Myocardial Infarction Occurrences	Number of Myocardial Infarction Occurrences		
226	CDMI	Char	Occurrences Myocardial Infarction	An acute myocardial infarction which occurred intraoperatively or within 30 days following surgery as manifested by one of the following: * Documentation of ECG changes indicative of acute MI (one or more of the following):<br>- ST elevation > 1 mm in two or more contiguous leads<br>- New left bundle branch<br>- New q-wave in two of more contiguous leads<br>* New elevation in troponin greater than 3 times upper level of the reference range in the setting of suspected myocardial ischemia * Physician diagnosis of myocardial infarction	Myocardial Infarction; No Complication	Definition revised or clarified from 2011
227	DCDMI	Num	Days from Operation until Myocardial Infarction Complication	Days from Operation until Myocardial Infarction Complication		-99 = Patient did not experience this complication at or before 30 days post operation
228	NOTHBLEED	Num	Number of Bleeding Transfusions Occurrences	Number of Bleeding Transfusions Occurrences		

Position #	Variable Name	Data Type	Variable Label	Variable Definition	Variable Options at Entry	Comments
229	OTHBLEED	Char	Occurrences Bleeding Transfusions	At least 1 unit of packed or whole red blood cells given from the surgical start time up to and including 72 hours postoperatively. If the patient receives shed blood, autologous blood, cell saver blood or pleurovac postoperatively, count this blood in terms of equivalent units. For a cell saver, every 500 ml's of fluid will equal 1 unit of packed cells. If there are less than 250 ml of cell saver, round down and report as 0 units. If there are 250 cc, or more of cell saver, round up to 1 unit. The blood may be given for any reason. If greater than 200 units, enter 200 units. Record the number of units given. Record the date the blood was initially started (intra-operatively or postoperatively).<br>Note: Intra-operative blood to prime the by-pass pump for CABG is not shed blood and should not be included as cell-saver blood.	Transfusions/Intraop/Postop; No Complication	Definition change from 2009
230	DOTHBLEED	Num	Days from Operation until Bleeding Transfusions Complication	Days from Operation until Bleeding Transfusions Complication		-99 = Patient did not experience this complication at or before 30 days post operation (One case which had a Bleeding Transfusion complication had an unknown date and thus the duration was set to -99)
231	NOTHGRAFL	Num	Number of Graft/Prosthesis/Flap Failure Occurrences	Number of Graft/Prosthesis/Flap Failure Occurrences		
232	OTHGRAFL	Char	Occurrences Graft/Prosthesis/FF	Mechanical failure of an extracardiac graft or prosthesis including myocutaneous flaps and skin grafts requiring return to the operating room, interventional radiology, or a balloon angioplasty within 30 days of the operation.	Graft/Prosthesis/Flap Failure; No Complication	
233	DOTHGRAFL	Num	Days from Operation until Graft/Prosthesis/Flap Failure Complication	Days from Operation until Graft/Prosthesis/Flap Failure Complication		-99 = Patient did not experience this complication at or before 30 days post operation
234	NOTHDVT	Num	Number of DVT/Thrombophlebitis Occurrences	Number of DVT/Thrombophlebitis Occurrences		
235	OTHDVT	Char	Occurrences DVT/Thrombophlebitis	New diagnosis of blood clot or thrombus within the venous system (superficial or deep) which may be coupled with inflammation and requires treatment.<br>Must be noted within 30 days after the principal operative procedure AND one of the following A or B below:<br>A.New Diagnosis of a [new] venous thrombosis (superficial or deep), confirmed by a duplex, venogram, CT scan, or any other definitive imaging modality (including direct pathology examination such as autopsy) AND the patient must be treated with anticoagulation therapy and/or placement of a vena cava filter or clipping of the vena cava, or the record indicates that treatment was warranted but there was no additional appropriate treatment option available.<br>B. As per (A) above, but the patient or decisionmaker has refused treatment. There must be documentation in the medical record of the [patient's] refusal of treatment.	DVT Requiring Therapy; No Complication	Definition revised or clarified from 2013
236	DOTHDVT	Num	Days from Operation until DVT/Thrombophlebitis Complication	Days from Operation until DVT/Thrombophlebitis Complication		-99 = Patient did not experience this complication at or before 30 days post operation
237	NOTHSYSEP	Num	Number of Sepsis Occurrences	Number of Sepsis Occurrences		

Position #	Variable Name	Data Type	Variable Label	Variable Definition	Variable Options at Entry	Comments
238	OTHSYSEP	Char	Occurrences Sepsis	Sepsis is a vast clinical entity that takes a variety of forms. The spectrum of disorders spans from relatively mild physiologic abnormalities to septic shock. The intent is to capture the patient whose physiology is compromised by an ongoing infectious process after surgery. Present at the time of surgery (PATOS) modifiers prevent patients from being counted as having complications if there is significant evidence that the sepsis or septic shock outcome was under way prior to the surgery performed. Please report the most significant level using the criteria below.<br>**1.Sepsis**: Sepsis is the systemic response to infection. Report this variable if the patient has two of the following clinical signs and symptoms of SIRS:<br>*Temp >38o C (100.4 o F) or < 36 o C (96.8 o F)<br>*HR >90 bpm<br>*RR >20 breaths/min or PaCO2 <32 mmHg(<4.3 kPa)<br>*WBC >12,000 cell/mm3, <4000 cells/mm3, or >10% immature (band) forms<br>*Anion gap acidosis: this is defined by either: [Na + K] – [Cl + HCO3 (or serum CO2)]. If this number is greater than 16, then an anion gap acidosis is present. Na – [Cl + HCO3 (or serum CO2)]. If this number is greater than 12, then an anion gap acidosis is present. *If anion gap lab values are performed at your facilities lab, ascertain which formula is utilized and follow guideline criteria.<br>And either A or B below:<br>A. One of the following:<br>*positive blood culture<br>*clinical documentation of purulence or positive culture from any site for which there is documentation noting the site as the acute cause of sepsis<br><br>B. The patient must meet SIRS criteria within 48 hours after the Principal Operative Procedure AND One of the following findings during the Principal Operative Procedure:<br>*Confirmed infarcted bowel requiring resection<br>*Purulence in the operative site<br>*Enteric contents in the operative site, or<br>*Positive intra-operative cultures<br>Guidance: if the patient meets criteria to assign preop sepsis, assign the risk factor; if the patient meets the criteria to assign postop sepsis, assign the occurrence and then assess for PATOS and assign if appropriate.<br>2. Septic Shock: Sepsis is considered severe when it is associated with organ and/or circulatory dysfunction. Report this variable if the patient has sepsis AND documented organ and/or circulatory dysfunction. Examples of organ dysfunction include: oliguria, acute alteration in mental status, acute respiratory distress. Examples of circulatory dysfunction include: hypotension, requirement of inotropic or vasopressor agents. Septic Shock is assigned when it appears to be related to Sepsis and not a Cardiogenic or Hypovolemic etiology.<br>Guidance: if the patient meets criteria to assign preop septic shock assign the risk factor; if the patient meets the criteria to assign postop septic shock assign the occurrence and then assess for PATOS and assign if appropriate.	Sepsis; No Complication	Definition revised or clarified from 2013
239	SEPSISPATOS	Char	Sepsis PATOS	If sepsis is noted as a postoperative outcome; select YES (for PATOS) if preoperative data are highly suggestive or suspicious of Sepsis being present at the time of surgery. If the record indicates that sepsis was present at some point preoperatively but fully and definitively resolved prior to the time of surgery, then PATOS should not be chosen.<br>Guidance: if postoperative “Sepsis” is assigned--only “PATOS Sepsis” can be assigned (provided that the patient meets criteria for PATOS Sepsis). Cannot assign “PATOS Septic shock” unless septic shock occurs postoperatively.	Yes; No	NULL = No Response Variable added in 2012
240	DOTHSYSEP	Num	Days from Operation until Sepsis Complication	Days from Operation until Sepsis Complication		-99 = Patient did not experience this complication at or before 30 days post operation
241	NOTHSESHOCK	Num	Number of Septic Shock Occurrences	Number of Septic Shock Occurrences		

Position #	Variable Name	Data Type	Variable Label	Variable Definition	Variable Options at Entry	Comments
242	OTHSESHOCK	Char	Occurrences Septic Shock	For Sepsis and Septic Shock within 30 days of the operation, please report the most significant level using the criteria that follow. Severe Sepsis/Septic Shock: Sepsis is considered severe when it is associated with organ and/or circulatory dysfunction. Report this variable if the patient has the clinical signs and symptoms of SIRS or sepsis **AND** documented organ and/or circulatory dysfunction. Examples of organ dysfunction include: oliguria, acute alteration in mental status, acute respiratory distress. Examples of circulatory dysfunction include: hypotension, requirement of inotropic or vasopressor agents. For the patient that had sepsis preoperatively, worsening of any of the above signs postoperatively would be reported as a postoperative sepsis.	Septic Shock; No Complication	
243	SEPSHOCKPATOS	Char	Septic Shock PATOS	If septic shock is noted as a postoperative occurrence; select YES (for PATOS) if preoperative data are highly suggestive or suspicious of Septic Shock being present at the time of surgery. If the record indicates that septic shock was present at some point preoperatively but fully and definitively resolved prior to the time of surgery, then PATOS should not be chosen.<br>Guidance: if postoperative "Septic shock" is assigned--only "PATOS septic shock" can be assigned (provided the patient meets criteria for PATOS septic shock). Cannot assign "PATOS sepsis" because septic shock	Yes; No	NULL = No Response Variable added in 2012
244	DOTHSESHOCK	Num	Days from Operation until Septic Shock Complication	Days from Operation until Septic Shock Complication		-99 = Patient did not experience this complication at or before 30 days post operation
245	PODIAG	Char	Post-op diagnosis (ICD 9)	The appropriate ICD-9-CM code corresponding to the condition noted as the postoperative diagnosis in the brief operative note, operative report, and/or after the return of the pathology reports are entered.		
246	PODIAGTX	Char	Post-op Diagnosis Text	Post-op Diagnosis text		
247	RETURNOR	Char	Return to OR	Returns to the operating room within 30 days include all major surgical procedures that required the patient to be taken to the surgical operating room for intervention of any kind. "Major surgical procedures" are defined as those cases in any and all surgical subspecialties that meet Program criteria for inclusion.	Yes; No	NULL= Unknown
248	DSDtoHD	Num	Days from Surgical Discharge (Acute Care Discharge) to Hospital Discharge	Days from Surgical Discharge to Hospital Discharge		Historical variable, no longer used
249	DOpertoD	Num	Days from Operation to Death	Days from Operation to Death		-99 = Patient did not die at or before 30 days post operation
250	DOptoDis	Num	Days from Operation to Discharge	Days from Operation to Discharge		-99 = Unknown
251	STILLINHOSP	Char	Still in Hospital > 30 Days	"Yes" is entered if patient has a continuous stay in the acute care setting > 30 days after the surgery. However, if the patient was discharged from the acute care setting, but remained in the hospital (rehab or hospice unit), then "NO" is entered, since the stay in the acute care setting was no longer continuous.	Yes; No	NULL = No Response Variable added in 2011
252	READMISSION	Char	Readmssion	"Yes" is entered for any readmission (to the same or another hospital), for any reason, within 30 days of the principal surgical procedure. The readmission has to be classified as an "inpatient" stay by the readmitting hospital, or reported by the patient/family as such.	Yes; No	Historical variable, no longer used NULL=Unknown
253	UNPLANREADMISSION	Char	Unplanned Readmission	"Yes" is entered for any unplanned readmission (to the same or another hospital) for a post operative occurrence likely related to the principal surgical procedure within 30 days of the procedure.	Yes; No	Historical variable, no longer used NULL=Unknown
254	REOPERATION	Char	Unplanned Reoperation	"Yes" is entered if the patient had an unplanned return to the operating room for a surgical procedure related to either the index or concurrent procedure performed. This return must be within the 30 day postoperative period. The return to the OR may occur at any hospital or surgical facility (i.e. your hospital or at an outside hospital). Note: This definition is not meant to capture patients who go back to the operating room within 30 days for a follow-up procedure based on the pathology results from the index or concurrent procedure. Examples: Exclude breast biopsies which return for re-excisions; insertion of port-a-caths for chemotherapy.	Yes; No	Historical variable, no longer used NULL=Unknown

Position #	Variable Name	Data Type	Variable Label	Variable Definition	Variable Options at Entry	Comments
255	REOPERATION1	Char	Unplanned Reoperation 1	"Yes" is entered if the patient had an unplanned return to the operating room for a surgical procedure, for any reason, within 30 days of the principal operative procedure. The return to the OR may occur at any hospital or surgical facility (i.e. your hospital or at an outside hospital).	Yes; No	Variable added in 2012
256	RETORPODAYS	Num	Days from principal operative procedure to Unplanned Reoperation 1	Days from principal operative procedure to Unplanned Reoperation 1		-99 = Patient did not experience Unplanned Reoperation 1 Variable added in 2012
257	REOPORCPT1	Char	Unplanned Reoperation 1 CPT	The CPT code for Unplanned Reoperation 1		NULL = No Response Variable added in 2012
258	RETORRELATED	Char	Unplanned Reoperation 1 related to principal operative procedure	Was the return to the OR for a postoperative occurrence likely related to the principal operative procedure? "Yes" is the default answer unless it is definitively indicated that the unplanned return to the OR is not related to the principal operative procedure.	Yes<br>No<br>Unknown	NULL = No Response Variable added in 2012 Definition revised in 2013
259	REOPORICD91	Char	Unplanned Reoperation 1 ICD-9	The ICD-9 code for Unplanned Reoperation 1		NULL = No Response Variable added in 2012
260	REOPERATION2	Char	Unplanned Reoperation 2	"Yes" is entered if the patient had an unplanned return to the operating room for a surgical procedure, for any reason, within 30 days of the principal operative procedure. The return to the OR may occur at any hospital or surgical facility (i.e. your hospital or at an outside hospital).	Yes; No	Variable added in 2012
261	RETOR2PODAYS	Num	Days from principal operative procedure to Unplanned Reoperation 2	Days from principal operative procedure to Unplanned Reoperation 2		-99 = Patient did not experience Unplanned Reoperation 2 Variable added in 2012
262	REOPOR2CPT1	Char	Unplanned Reoperation 2 CPT	The CPT code for Unplanned Reoperation 2		NULL = No Response Variable added in 2012
263	RETOR2RELATED	Char	Unplanned Reoperation 2 related to principal operative procedure	Was the return to the OR for a postoperative occurrence likely related to the principal operative procedure? "Yes" is the default answer unless it is definitively indicated that the unplanned return to the OR is not related to the principal operative procedure.	Yes<br>No<br>Unknown	NULL = No Response Variable added in 2012 Definition revised in 2013
264	REOPOR2ICD91	Char	Unplanned Reoperation 2 ICD-9	The ICD-9 code for Unplanned Reoperation 2		NULL = No Response Variable added in 2012
265	REOPERATION3	Char	More than 2 unplanned reoperations	"Yes" is entered if there were more than two unplanned re-operations for a post operative occurrence likely related to the principal surgery within 30 days.	Yes; No	Variable added in 2012
266	READMISSION1	Char	Any Readmission 1	"Yes" is entered if the patient had any readmission (to the same or another hospital), for any reason, within 30 days of the principal surgical procedure. The readmission has to be classified as an "inpatient" stay by the readmitting hospital, or reported by the patient/family as such.	Yes; No	Variable added in 2012
267	READMPODAYS1	Num	Days from principal operative procedure to Any Readmission 1	Days from principal operative procedure to Any Readmission 1		-99 = Patient did not experience Any Readmission 1 Variable added in 2012
268	UNPLANNEDREADMISSION1	Char	Unplanned Readmission 1	"Yes" is entered if Any Readmission 1 was unplanned at the time of the principal procedure.	Yes; No	NULL = No Response Variable added in 2012
269	READMRELATED1	Char	Unplanned Readmission 1 likely related to the principal procedure	"Yes" is entered if Unplanned Readmission 1 (to the same or another hospital) was for a postoperative occurrence likely related to the principal surgical procedure within 30 days of the procedure.	Yes; No	NULL = No Response Variable added in 2012
270	READMSUSPREASON1	Char	Readmission related suspected reason 1	The primary suspected reason for the readmission if it is likely related to the principal operating procedure	Superficial SSI<br>Deep Incisional SSI<br>Organ/Space SSI<br>Wound Disruption<br>Pneumonia<br>Unplanned Intubation<br>Pulmonary Embolism<br>On Ventilator > 48 hours<br>Progressive Renal Insufficiency<br>Acute Renal Failure<br>Urinary Tract Infection<br>CVA<br>Cardiac Arrest Requiring CPR<br>Myocardial Infarction<br>Bleeding Requiring Transfusion (72h of surgery start time)<br>DVT Requiring Therapy<br>Sepsis<br>Septic Shock<br>Other (list ICD 9 code)	NULL = No Response Variable added in 2012

Position #	Variable Name	Data Type	Variable Label	Variable Definition	Variable Options at Entry	Comments
271	READMUNRELSUSP1	Char	Readmission unrelated suspected reason 1	The primary suspected reason for the readmission if it is likely unrelated to the principal operating procedure	Superficial SSI<br>Deep Incisional SSI<br>Organ/Space SSI<br>Wound Disruption<br>Pneumonia<br>Unplanned Intubation<br>Pulmonary Embolism<br>On Ventilator > 48 hours<br>Progressive Renal Insufficiency<br>Acute Renal Failure<br>Urinary Tract Infection<br>CVA<br>Cardiac Arrest Requiring CPR<br>Myocardial Infarction<br>Bleeding Requiring Transfusion (72h of surgery start time)<br>DVT Requiring Therapy<br>Sepsis<br>Septic Shock<br>Other (list ICD 9 code)	NULL = No Response Variable added in 2013
272	READMRELICD91	Char	Readmission related ICD-9 code 1	The ICD-9 code for the suspected reason if 'Other' is chosen and the readmission is likely related to the principle operating procedure		NULL = No Response Variable added in 2012
273	READMUNRELICD91	Char	Readmission unrelated ICD-9 code 1	The ICD-9 code for the suspected reason if 'Other' is chosen and the readmission is likely unrelated to the principle operating procedure		NULL = No Response Variable added in 2013
274	READMISSION2	Char	Any Readmission 2	See "Any Readmission 1"	Yes; No	Variable added in 2012
275	READMPODAYS2	Num	Days from principal operative procedure to Any Readmission 2	See "Days from principal operative procedure to Any Readmission 1"		-99 = Patient did not experience Any Readmission 2 Variable added in 2012
276	UNPLANNEDREADMISSION2	Char	Unplanned Readmission 2	See "Unplanned Readmission 1"	Yes;No	NULL = No Response Variable added in 2012
277	READMRELATED2	Char	Unplanned Readmission 2 likely related to the principal procedure	See "Unplanned Readmission 1 likely related to the principal procedure"	Yes;No	NULL = No Response Variable added in 2012
278	READMSUSPREASON2	Char	Readmission related suspected reason 2	See "Readmission related suspected reason 1"	See "Readmission related suspected reason 1"	NULL = No Response Variable added in 2012
279	READMUNRELSUSP2	Char	Readmission unrelated suspected reason 2	See "Readmission unrelated suspected reason 1"	See "Readmission unrelated suspected reason 1"	NULL = No Response Variable added in 2013
280	READMRELICD92	Char	Readmission related ICD-9 code 2	See "Readmission related ICD-9 code 1"		NULL = No Response Variable added in 2012
281	READMUNRELICD92	Char	Readmission unrelated ICD-9 code 2	See "Readmission unrelated ICD-9 code 1"		NULL = No Response Variable added in 2013
282	READMISSION3	Char	Any Readmission 3	See "Any Readmission 1"	Yes; No	Variable added in 2012
283	READMPODAYS3	Num	Days from principal operative procedure to Any Readmission 3	See "Days from principal operative procedure to Any Readmission 1"		-99 = Patient did not experience Any Readmission 3 Variable added in 2012
284	UNPLANNEDREADMISSION3	Char	Unplanned Readmission 3	See "Unplanned Readmission 1"	Yes;No	NULL = No Response Variable added in 2012
285	READMRELATED3	Char	Unplanned Readmission 3 likely related to the principal procedure	See "Unplanned Readmission 1 likely related to the principal procedure"	Yes; No	NULL = No Response Variable added in 2012
286	READMSUSPREASON3	Char	Readmission related suspected reason 3	See "Readmission related suspected reason 1"	See "Readmission related suspected reason 1"	NULL = No Response Variable added in 2012
287	READMUNRELSUSP3	Char	Readmission unrelated suspected reason 3	See "Readmission unrelated suspected reason 1"	See "Readmission unrelated suspected reason 1"	NULL = No Response Variable added in 2013
288	READMRELICD93	Char	Readmission related ICD-9 code 3	See "Readmission related ICD-9 code 1"		NULL = No Response Variable added in 2012
289	READMUNRELICD93	Char	Readmission unrelated ICD-9 code 3	See "Readmission unrelated ICD-9 code 1"		NULL = No Response Variable added in 2013
290	READMISSION4	Char	Any Readmission 4	See "Any Readmission 1"	Yes; No	Variable added in 2012
291	READMPODAYS4	Num	Days from principal operative procedure to Any Readmission 4	See "Days from principal operative procedure to Any Readmission 1"		-99 = Patient did not experience Any Readmission 4 Variable added in 2012
292	UNPLANNEDREADMISSION4	Char	Unplanned Readmission 4	See "Unplanned Readmission 1"	Yes; No	NULL = No Response Variable added in 2012
293	READMRELATED4	Char	Unplanned Readmission 4 likely related to the principal procedure	See "Unplanned Readmission 1 likely related to the principal procedure"	Yes; No	NULL = No Response Variable added in 2012
294	READMSUSPREASON4	Char	Readmission related suspected reason 4	See "Readmission related suspected reason 1"	See "Readmission related suspected reason 1"	NULL = No Response Variable added in 2012

Position #	Variable Name	Data Type	Variable Label	Variable Definition	Variable Options at Entry	Comments
295	READMUNRELSUSP4	Char	Readmission unrelated suspected reason 4	See "Readmission unrelated suspected reason 1"	See "Readmission unrelated suspected reason 1"	NULL = No Response Variable added in 2013
296	READMRELICD94	Char	Readmission related ICD-9 code 4	See "Readmission related ICD-9 code 1"		NULL = No Response Variable added in 2012
297	READMUNRELICD94	Char	Readmission unrelated ICD-9 code 4	See "Readmission unrelated ICD-9 code 1"		NULL = No Response Variable added in 2013
298	READMISSION5	Char	Any Readmission 5	See "Any Readmission 1"	Yes; No	Variable added in 2012
299	READMPODAYS5	Num	Days from principal operative procedure to Any Readmission 5	See "Days from principal operative procedure to Any Readmission 1"		-99 = Patient did not experience Any Readmission 5 Variable added in 2012
300	UNPLANNEDREADMISSION5	Char	Unplanned Readmission 5	See "Unplanned Readmission 1"	Yes; No	NULL = No Response Variable added in 2012
301	READMRELATED5	Char	Unplanned Readmission 5 likely related to the principal procedure	See "Unplanned Readmission 1 likely related to the principal procedure"	Yes; No	NULL = No Response Variable added in 2012
302	READMSUSPREASON5	Char	Readmission related suspected reason 5	See "Readmission related suspected reason 1"	See "Readmission related suspected reason 1"	NULL = No Response Variable added in 2012
303	READMUNRELSUSP5	Char	Readmission unrelated suspected reason 5	See "Readmission unrelated suspected reason 1"	See "Readmission unrelated suspected reason 1"	NULL = No Response Variable added in 2013
304	READMRELICD95	Char	Readmission related ICD-9 code 5	See "Readmission related ICD-9 code 1"		NULL = No Response Variable added in 2012
305	READMUNRELICD95	Char	Readmission unrelated ICD-9 code 5	See "Readmission unrelated ICD-9 code 1"		NULL = No Response Variable added in 2013

VARIABLE ADDED IN 2011
VARIABLE ADDED IN 2012
VARIABLE ADDED IN 2013

ACS NSQIP®

AMERICAN COLLEGE OF SURGEONS
Inspiring Quality:
Highest Standards, Better Outcomes

100+years

© 2014 American College of Surgeons
All rights reserved
